# Predicting adsorption selectivities from pure gas isotherms for gas mixtures in metal–organic frameworks†

**DOI:** 10.1039/c9sc03008e

**Published:** 2019-12-06

**Authors:** Arpan Kundu, Kaido Sillar, Joachim Sauer

**Affiliations:** Humboldt Universität zu Berlin, Institut für Chemie Unter den Linden 6 10099 Berlin Germany js@chemie.hu-berlin.de; University of Tartu, Institute of Chemistry Ravila 14a 50411 Tartu Estonia

## Abstract

We perform Grand Canonical Monte Carlo simulations on a lattice of Mg^2+^ sites (GCMC) for adsorption of four binary A/B mixtures, CH_4_/N_2_, CO/N_2_, CO_2_/N_2_, and CO_2_/CH_4_, in the metal–organic framework Mg_2_(2,5-dioxidobenzedicarboxylate), also known as CPO-27–Mg or Mg–MOF-74. We present a mean field co-adsorption isotherm model and show that its predictions agree with the GCMC results if the same quantum chemical *ab initio* data are used for Gibbs free energies of adsorption at the individual sites and for lateral interaction energies between the same, A⋯A and B⋯B, and unlike, A⋯B, adsorbed molecules. We use both approaches to test the assumption underlying Ideal Adsorbed Solution Theory (IAST), namely approximating A⋯B interaction energies as the arithmetic mean of A⋯A and B⋯B interaction energies. While IAST works well for mixtures with weak lateral interactions, CH_4_/N_2_ and CO/N_2_, the deviations are large for mixtures with stronger lateral interactions, CO_2_/N_2_ and CO_2_/CH_4_. Motivated by the theory of London dispersion forces, we propose use of the geometric mean instead of the arithmetic mean and achieve substantial improvements. For CO_2_/CH_4_, the lateral interactions become anisotropic. To include this in the geometric mean co-adsorption model, we introduce an anisotropy factor. We propose a protocol, named co-adsorption mean field theory (CAMT), for co-adsorption selectivity prediction from known (experiment or simulation) pure component isotherms which is similar to the IAST protocol but uses the geometric mean to approximate mixed pair interaction energies and yields improved results for non-ideal mixtures.

## Abstract 2

A new mixing rule (geometric mean) is proposed with substantial improvements compared to the widely used ideal adsorbed solution theory for adsorbates with strong lateral interactions.

## Graphical abstract

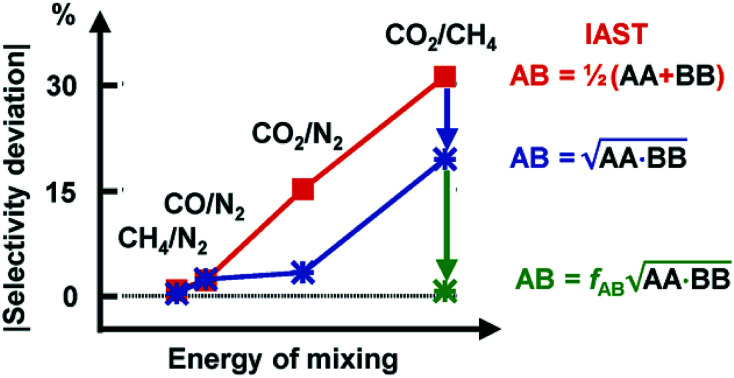

## 1. Introduction

Porous media with well-defined pore structures have a high potential for the separation of gases by selective adsorption. Classical crystalline microporous materials, zeolites, are used since decades for such tasks, *e.g.*, for air separation.^1^ With the synthesis of metal–organic frameworks (MOFs),^2,3^ new materials have become available with high adsorption capacities and tunable adsorption properties.^4–6^ Specifically, they have high potential for storage of energy carrying molecules (H_2_ and CH_4_)^7,8^ and for separation processes, such as CO_2_ and N_2_ removal for natural gas upgrade and CO_2_ capture from flue gas.^9–11^ Substituting natural gas for coal and post-combustion CO_2_ capture and storage (CCS) are key technologies to mitigate the environmental impact of burning fossil fuels.^12^

The selection and rational design of improved materials with optimized properties for a specific separation target requires reliable predictions of co-adsorption isotherms and adsorption selectivities. Because isotherm measurements are much more demanding for mixtures than for single components and require specific equipment,^13,14^ more than 50 years after its invention, in the vast majority of cases, Ideal Adsorbed Solution Theory (IAST)^15^ is still used to predict co-adsorption isotherms for gas mixtures from pure gas data.^16,17^ Also when simulation methods are used, the availability of methods for predicting mixture isotherms from pure components will speed up computational screening for optimal materials in separations, *e.g.*, ref. 18.

IAST assumes that mixture components behave like an ideal solution in the adsorbed phase – an approximation that is not always valid, for example when one component adsorbs more strongly than the other^19^ or is of very different size than the other.^20^ The ideal behavior of the adsorbed phase implies that the mixing energy is zero, which also defines the underlying approximation for the lateral interactions – the mixing energy can be zero only if the intermolecular interactions between the molecules of unlike components, *E*_AB_, are the average (arithmetic mean, AM) of the interactions between molecules of the individual mixture components A and B,^21,22^1
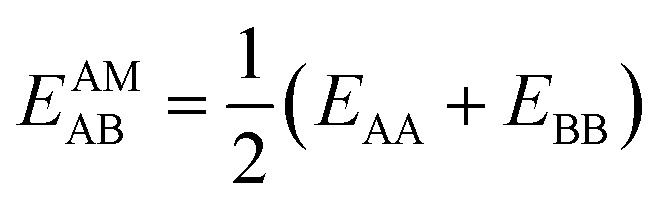


Here, we propose an improved method for predicting mixture isotherms from pure gas data that approximate the interaction energy between unlike molecules as the geometric mean (GM) of the interactions between molecules of the individual components,2
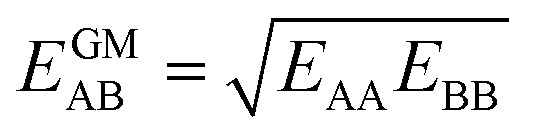
which is also known as the Berthelot combination rule^23^ and used for *C*_6_ dispersion parameters with many force fields, see, *e.g.*, ref. 24 and 25 for MOF force fields.

There are other ways of dealing with non-ideal mixing behavior, *e.g.*, Real Adsorbed Solution Theory (RAST)^26–28^ makes use of activity coefficients to take lateral interactions into account, but requires experimental or simulated co-adsorption data, whereas we focus here on predictions based on pure gas components only. RAST is typically applied to mixtures with strong adsorbate–adsorbate interactions, comparable in strength with adsorbate–surface interactions, *e.g.*, water–alcohol mixtures on microporous silica.^28,29^ Systems with strong lateral interactions are beyond the scope of our study.

We consider four binary gas mixtures: CO_2_/CH_4_ and N_2_/CH_4_ relevant for the natural gas upgrade, CO_2_/N_2_ relevant for flue gas separation for CCS, and CO/N_2_ for removal of toxic CO from gas mixtures (*e.g.*, burnt air) which might be relevant for gas mask applications and syngas production and purification. As an adsorbent, we consider Mg_2_(dobdc)^30–32^ (dobdc^4−^ = 2,5-dioxidobenzendicarboxylate), a MOF, also known as CPO-27–Mg and Mg–MOF-74, that is considered especially promising for CO_2_ adsorption because of its high concentration of accessible strong adsorption sites, “under-coordinated” (five-fold coordinated) Mg^2+^ ions. To these sites, CH_4_, N_2_, CO, and CO_2_ bind with 26, 29, 39, and 46 kJ mol^−1^, respectively (Table 1). Comparatively, the average lateral adsorbate–adsorbate energies are small, −0.55, −0.35, −0.34, and −2.81 kJ mol^−1^, respectively; only for CO_2_, they exceed the thermal energy at 298 K (−2.5 kJ mol^−1^).

**Table 1:** Energies, Δ*E*_A_ at 0 K,^a^ and Gibbs free energies of adsorption, Δ*G*_A_, at 298 K, for adsorbate A, as well as lateral interaction energies, *E*_AA_, from *ab initio* calculations, compared to parameters from mean-field fitting of *ab initio* GCMC isotherms using two procedures: (i) adsorption constants (non-linear fit)^b^ and (ii) free energies (linear fit).^c^ Energies in kJ mol^−1^ and the equilibrium constant, *K*, in 1 atm^−1^

		*Ab initio* d		MF fit of ads. constant			MF fit of free energy	
A	Δ*E*_A_	Δ*G*_A_	*E*_AA_ = *E*^av^_AA_	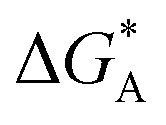 e	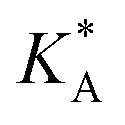	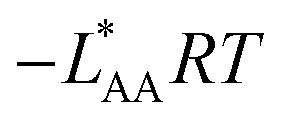 f	Δ*G*_A_	−*L*_AA_*RT*^f^
CO	−39.0	−1.11	−0.34	−1.11	1.5682	−0.34	−1.11	−0.33
N_2_	−29.2	4.16	−0.35	4.15	0.1872	−0.33	4.16	−0.32
CH_4_	−25.8	3.90	−0.55	3.87	0.2097	−0.46	3.89	−0.46
CO_2_	−45.9	−9.22	−2.81	−9.38	44.14	−3.24^g^	−9.25	−2.70

a Zero point vibrational energy contributions are included.

b Used for IAST, Section 4.1.

c Used for CMFfit, Section 4.2.

d Ref. 34–36.

e Calculated using eqn (21) from 
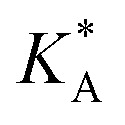
.

f See eqn (22), –*L*_AA_*RT* is comparable with *E*_AA_ because *N* = 2 and *E*^av^_AA_ = *E*_AA_ as there is no anisotropy in pure gas lateral interactions.

g After fitting with continued fraction representation, −2.63 kJ mol^−1^, see Section S6 in the ESI.

Although with increasing pressure also the adsorption sites at the dobdc linker molecules will become populated (our previous study^33^ on pure CO_2_ adsorption has shown that this will be the case for pressures higher than 0.05 atm) the present study considers a homogeneous lattice of Mg^2+^ adsorption sites as a model that captures essential features of MOFs with open metal ion sites.

For the CH_4_/N_2_, CO/N_2_, CO_2_/N_2_, and CO_2_/CH_4_ mixtures, we first predict co-adsorption isotherms and adsorption selectivities from quantum chemical *ab initio* calculations employing two methods:

(i) Grand Canonical Monte Carlo (GCMC) simulations on a lattice of adsorption sites which use Gibbs free energies of adsorption as input for gas molecules at isolated individual sites and calculate the interaction energies for each configuration explicitly,^33^ both generated from quantum chemical *ab initio* calculations with chemical accuracy (4 kJ mol^−1^ or better).^34–36^ These results will serve as a benchmark for any other method used in this study.

(ii) Competitive mean-field (CMF) isotherm model that has clearly separated and physically meaningful parameters for molecule-surface and molecule–molecule (lateral) interactions.^34–36^ Before, we have used the mean field (Bragg–Williams) model for pure gases;^34–36^ here we extend it to mixture co-adsorption by including the interaction energy term between molecules of different gases in addition to the lateral interaction energies between the same gas molecules.^37^

For pure gases, we have shown before that MF theory which assumes an average value for lateral interaction energies not only yields isotherms in close agreement with experiments,^34–36^ but also in close agreement with the results of GCMC simulations on a lattice of adsorption sites.^33^ Here, for mixtures, we find the same level of agreement between the results of the analytical CMF equations and the benchmark GCMC simulations with the same *ab initio* data as the input.

Next, we use both GCMC and CMF co-adsorption isotherms and selectivities to test the AM approximation and the CMF results to test both the AM and GM approximations. For the adsorbed mixtures with very weak lateral interactions, CH_4_/N_2_ and CO/N_2_, we find that mixing energies are indeed negligible (less than 0.2 kJ mol^−1^) and, consequently, AM mixing is a good approximation and GM mixing has no advantage. For mixtures with stronger lateral interactions, CO_2_/N_2_ and CO_2_/CH_4_, isotherms obtained with AM mixing deviate substantially from the GCMC benchmarks and GM mixing is always an improvement, whereas for CO_2_/N_2_ with GM close agreement with the GCMC benchmark is reached, and for CO_2_/CH_4_, an “anisotropy” factor is needed to account for different mixed pair interactions in different directions of the adsorbate layer.

Based on the insight gained, we propose an improved protocol for predicting co-adsorption selectivities from measured (or calculated) pure gas adsorption isotherms. As with the classical IAST protocol, the starting point is fitting the pure component adsorption data with an isotherm model. For this we use the mean-field (Bragg–Williams) isotherm model. Co-adsorption data are then obtained with the CMF equations, using different mixing rules for unlike pair interactions. When AM mixing is used, it reproduces IAST,^38^ whereas improved results are obtained when GM mixing is applied. The final step is inclusion of an anisotropy factor which needs an atomistic model of adsorption structures. This is not an obstacle because structural optimization using force fields or density functional theory (DFT) has become routine.

This article is organized as follows. Section 2 describes *ab initio* calculations of Gibbs free energies for adsorption of N_2_, CO, CO_2_, and CH_4_ on Mg^2+^ sites and of molecule–molecule interaction energies in the adsorbate layers. Section 3 presents *ab initio* predictions of co-adsorption isotherms and selectivities using GCMC simulations and CMF equations including tests of the AM and GM approximations. Section 4 presents our geometric mean model for predicting co-adsorption isotherms and selectivities from fitted pure gas data as an alternative to IAST.

## 2. *Ab initio* calculation of adsorption structures and energies

The *ab initio* Gibbs free energies of adsorption for the gas components A and B, Δ*G*_A_ and Δ*G*_B_, respectively, are taken from our previous studies^34–36^ and listed in Table 1. Chemical accuracy has been achieved for electronic energies by employing a quantum chemical hybrid method that uses Møller–Plesset second order perturbation theory (MP2)^39^ at the adsorption site and DFT+dispersion (PBE+D2) for the full periodic structures.^40–43^ For a smaller model of the adsorption site calculations are performed with Coupled Cluster theory, CCSD(T).^39^ Thermal effects and entropies for CO and N_2_ adsorption are obtained from vibrational partition functions with the anharmonic vibrational energies calculated for each vibrational mode separately.^44–46^ For adsorbed CH_4_ the partition function is calculated assuming that CH_4_ has retained all its rotational degrees of freedom upon adsorption^34^ while for CO_2_ only the vibrational mode that corresponds to the hindered rotation of CO_2_ is approximated by a one-dimensional free rotation.^36^

**Fig. 1:**
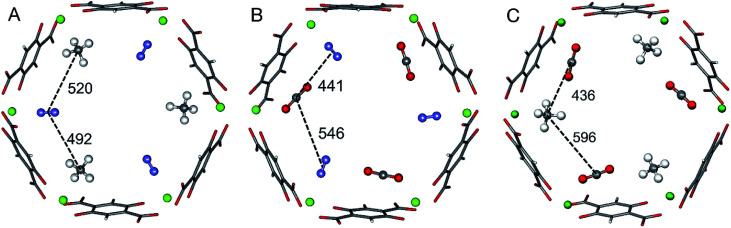
Adsorbate structures of CH_4_/N_2_ (A) CO_2_/N_2_ (B), and CO_2_/CH4 (C) mixtures in Mg_2_(dobdc). Distances between the centers of mass of the adsorbed molecules are given in pm.

For three of the four binary mixtures, Fig. 1 shows the adsorption structures with full occupation of the Mg^2+^ sites taken from previous PBE+D2 structural optimizations under periodic boundary conditions, see ref. 33–36. The figure shows the relevant interactions in the *a*,*b*-plane of the hexagonal pore. The distances in the *z*-direction, a unit cell length of 689 pm, are much larger and the corresponding interactions can be neglected. For pairs of adsorbed molecules taken from these periodic structures the lateral interaction energies are calculated using Coupled Cluster (CC) theory with complete basis set (CBS) extrapolation (CCSD(T)/CBS(D,T)). The pairs are isolated, *i.e.*, the framework is not present in these calculations.

For the CO_2_/CH_4_ mixture the “mixed” term for the lateral interaction energies between unlike molecules is taken from our previous work,^33^ whereas for the other mixtures the CO⋯N_2_, CH_4_⋯N_2_, and CO_2_⋯N_2_ interaction energies are calculated in this work. Table 2 shows the results. All Mg^2+^ adsorption sites are equivalent and for adsorbed pure gases all interactions between adsorbed molecules are also equivalent, but with mixed adsorbates “symmetry breaking” occurs. The distances and the interaction energies between neighboring unlike molecules depend on the direction of the interactions, *i.e.* they are anisotropic. When a pair of unlike molecules gets closer in one direction, the lateral interaction between them gets stronger, whereas the interactions with a molecule in opposite direction gets more distant with a weaker interaction, see Fig. 1 and Table 2.

**Table 2:** Lateral adsorbate–adsorbate interaction energies, *E*_AB_, between gas molecules of different (A⋯B) components. The average (av), the arithmetic mean (AM), and the geometric mean (GM) of the corresponding pure gas values are given together with the energy of mixing, Δ*E*_mix_. The average lateral interaction energies (−*L*_AB_*RT*) and energies of mixing (−*RT*Δ*L*_mix_) obtained by fitting are also given; all in kJ mol^−1^

A/B		CH_4_/N_2_	CO/N_2_	CO_2_/N_2_	CO_2_/CH_4_
*E*_AB_	Short	−0.54	−0.81	−1.68	−1.07
	Long	−0.40	−0.14	−0.48	−0.22
*E* ^av^_AB_(*ab initio*)	Average	−0.47	−0.48	−1.08	−0.65
*E* ^AM^_AB_ a		−0.45	−0.35	−1.58	−1.68
Δ*E*_mix_^b^		−0.02	−0.13	0.50	1.04
*E* ^GM^_AB_		−0.44	−0.34	−0.99	−1.24 (−0.62)^c^
Δ*E*^GM^_mix_		0.01	0.00	0.59	0.44 (1.06)^d^
−*RT L*^AM^_AB_ e	Fit	−0.39	−0.33	−1.51	−1.58
−*RT L*^GM^_AB_	Fit	−0.38	−0.32	−0.93	−1.11 (−0.56)^f^
−*RT* Δ*L*^GM^_mix_ g	Fit	0.01	0.00	0.58	0.47 (1.02)^h^

a Corresponds to IAST.

b See eqn (9).

c Effective lateral interaction energy, *E*^GM^_AB_/2, see Section 3.5, eqn (16) and (17).

d Effective energy of mixing.

e Corresponds to IAST; mixing energy is 0 kJ mol^−1^.

f Effective *L*_AB_-parameter, *L*^GM^_AB_/2, see Section 4.4, eqn (30).

g *L* parameter for mixing, defined as Δ*L*_mix_ = *L*_AB_−(*L*_AA_ + *L*_BB_)/2; see Table 1 for *L*_AA_ and *L*_BB_ parameters obtained from linear MF fitting.

h In parenthesis, effective energy of mixing after considering *f*_AB_ = 0.5.

## 3. *Ab initio* prediction of co-adsorption isotherms and selectivities

### 3.1. Grand canonical Monte Carlo simulation on a lattice of adsorption sites

We rely on our GCMC method on a lattice of sites^33^ to generate co-adsorption isotherms for the CH_4_/N_2_, CO/N_2_, CO_2_/N_2_, and CO_2_/CH_4_ mixtures. For pure gas (CO_2_, CH_4_) adsorption we have shown before that this method is reliable and yields isotherms in very close agreement with the experimental results when accurate (±1 kJ mol^−1^) *ab initio* Gibbs free energies are used as the input.^33,36^ The same can be expected for mixtures, and the lattice GCMC method has the additional advantage that the effect of different approximations for the lateral interactions can be tested.

We consider a lattice that contains *M* Mg^2+^ adsorption sites (neglecting adsorption on the weaker linker sites). Each site can adsorb a gas molecule, either A or B from a binary gas mixture. For a particular lattice gas configuration *i* containing *M*_A_ and *M*_B_ adsorbed molecules of components A and B, respectively, there are *M*_AA_, *M*_BB_, and *M*_AB_ interacting A⋯A, B⋯B, and A⋯B pairs, respectively, and the lattice gas Hamiltonian, *H*_*i*_, which represents the total free energy of the configuration *i* is

3 *H*_*i*_ = Δ*G*_A_*M*_A_ + Δ*G*_B_*M*_B_ + *E*_AA_*M*_AA_ + *E*_BB_*M*_BB_ + *E*_AB_*M*_AB_; *i* = 1,2,…,3^*M*^

Here, Δ*G*_A_ and Δ*G*_B_, are the *ab initio* Gibbs free energies of adsorption for the gas components A and B, respectively, and *E*_AA_, *E*_BB_, and *E*_AB_ denote the *ab initio* lateral interaction energies for a pair of adsorbed A⋯A, B⋯B and A⋯B molecules, respectively. The adsorption free energies and the lateral interaction energies are taken from our previous studies^34–36^ and are listed in Table 1. The entropic contributions to the lateral interactions are not included in this Hamiltonian. Compared to the contribution for the interaction with Mg^2+^ sites included in Δ*G*_A_ (−25.8 kJ mol^−1^ at 298 K, see Table 1), they are neglectable (about 0.5 kJ mol^−1^) as our previous calculations for pure CH_4_ adsorption have shown,^34^ and within the uncertainty limits of both the *ab initio* energy calculations and the DFT-D calculations of vibrational wavenumbers.

As mentioned above, the lateral interaction energies are calculated for isolated pairs, *i.e.*, the framework is not present in these calculations. Taking these energies from total energies for the full periodic structures would not be consistent with the use of constant Δ*G*_A_ and Δ*G*_B_ values in eqn (3), see also the ESI, Section S4.†

**Scheme 1:**
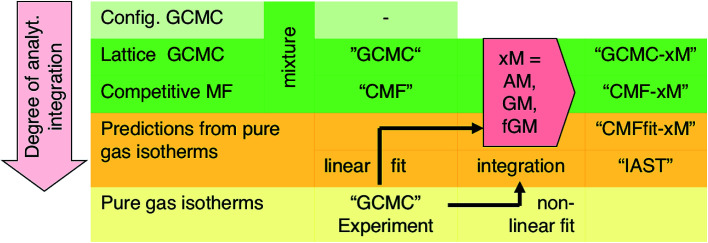
Overview of different simulation methods used in this study.

For a lattice gas model of moderate size with, *e.g.*, *M* = 6 × 100 sites as used here, the number of possible configurations becomes enormously large – 2^*M*^ and 3^*M*^ for a pure gas and a binary mixture, respectively. Hence, adsorption isotherms cannot be calculated analytically from the partition function. A GCMC simulation on this lattice gas model samples only the important configurations at a constant chemical potential and temperature. From these configurations, adsorption isotherms are calculated as the ensemble average of the number of molecules adsorbed.^33^ Unlike the mean field approach introduced in Section 3.2, our GCMC simulations on a lattice of adsorption sites treat all the lateral interactions exactly, and we will refer to them as “GCMC” in the following, see Scheme 1.

Commonly, GCMC simulations are not performed on a lattice of sites but directly in configuration space which requires several orders of magnitude more energy evaluations. This is not affordable with *ab initio*, not even with DFT potential energy surfaces, and typically force fields are used. GCMC calculations and similar simulation techniques have been used with force fields before to test the accuracy of IAST.^16,18,29,47–50^

### 3.2. Mean-field approximation for a binary adsorbed mixture with lateral interactions

Since GCMC simulations are computationally very demanding, even if done on a lattice of sites, the question emerges if an average value for the lateral interaction energies can be used instead of their explicit evaluation for each of the configurations sampled. For pure gases this is a well-established approximation that leads to the analytical Bragg–Williams isotherm equations,^51^ which we have used in the past with much success for *ab initio* predictions.^33–36^

Here, we extend the mean-field approximation to binary gas mixtures with components A and B. For the general case with many (*m*) components, see the ESI, Section S1.† The coverage dependent Gibbs free energy for component A in the mixture is4
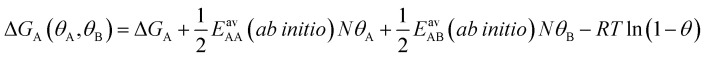
There is a corresponding expression for Δ*G*_B_. The four quantities in eqn (4) that determine the surface coverage of mixture components A and B, *θ*_A_ and *θ*_B_, respectively, are: (i) The Gibbs free energy of adsorption of an A molecule to an isolated adsorption site, Δ*G*_A_. (ii) and (iii) The lateral interaction energies between the same (A⋯A, pure gas term) and different (A⋯B, mixing term) mixture components, *E*^av^_AA_ and *E*^av^_AB_, respectively. They are calculated from the *ab initio* interaction energies for all respective pairs AX = AA and AB, respectively,5
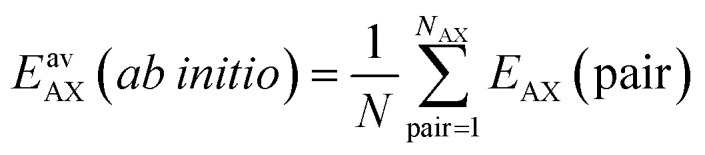
with *N* denoting the topological number of A⋯B neighbors, *i.e.*, 2 for the adsorbates shown in Fig. 1. The multiplier 1/2 in eqn (4) avoids double counting of interaction energies. The interaction energies *E*_AX_ are evaluated for isolated pairs as mentioned above, see also the ESI, Section S4.† (iv) The configurational entropy (Langmuir term, the last term in eqn (4)) depends on the total coverage *θ* = *θ*_A_ + *θ*_B_.

From eqn (4) we obtain the single component adsorption equilibrium constants for the mixture (see also Section S1 in the ESI†)6
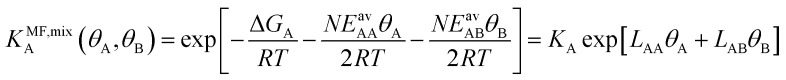
Here, *K*_A_ = exp[−Δ*G*_A_/*RT*] is the zero-coverage equilibrium constant of adsorption for mixture component A and adsorbate–adsorbate interactions may be described by the *L*-parameters,7
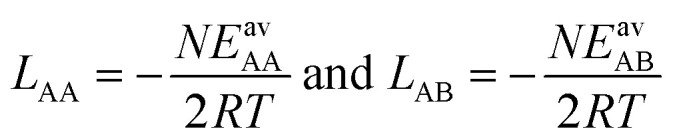
where *N* is the number of neighbors.

The surface coverage is obtained as8
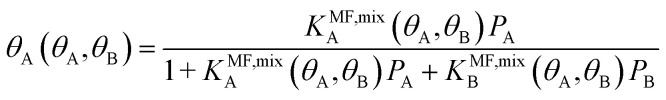
where *P*_c_ is the partial pressure of the component c in the gas mixture which defines the “competitive mean field” (CMF) model of co-adsorption. We will refer to mixture isotherms obtained this way as “CMF”, see Scheme 1.

Under the limiting conditions of no lateral interactions, *i.e.*, *E*^av^_AA_(*ab initio*) = *E*^av^_AB_(*ab initio*) = 0, eqn (8) reduces to the competitive Langmuir model of co-adsorption (*K*^MF,mix^_c_ = *K*_c_ = exp[−Δ*G*_c_/*RT*], c = A, B). When lateral interactions are non-negligible, *K*^MF,mix^_A_ and *K*^MF,mix^_B_ become a function of the coverages and, hence, eqn (8) turns into a self-consistent equation where the coverage of each gas component, *θ*_A or B_ = *f*(*θ*_A_,*θ*_B_), is also a function of itself. We solve this CMF model using an iterative process with coverages from the competitive Langmuir model as an initial guess. Usually, a few iterations are sufficient to yield converged surface coverages (see also Fig. S1 in the ESI†).

### 3.3. Different combination rules for mixed interaction energies

The ideal behavior of the adsorbed phase assumed by IAST implies that the mixing energy,9
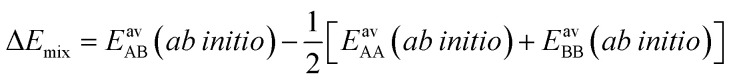
is zero, which can be the case only if the lateral interactions between the molecules of unlike components, *E*_AB_, are the average (arithmetic mean, AM, eqn (1)) of the interactions between molecules of the individual mixture components A and B.^21,22^ As improvement compared to the AM, here we propose to approximate the interaction energy between unlike molecules as the geometric mean (GM) of the interactions between molecules of the individual components, eqn (2). It is derived from the London formula for the *C*_6AB_ parameters (*α* – polarizability, *I* – ionization potential)10
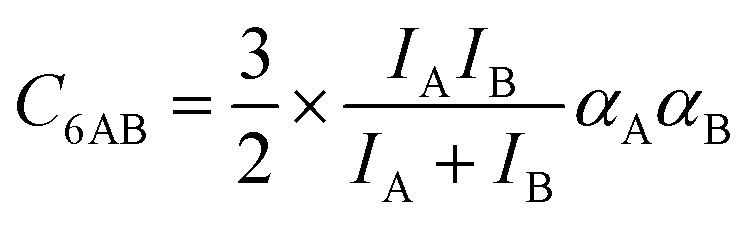
which yields *C*_6AB_ dispersion parameters between components A and B as the GM of *C*_6AA_ and *C*_6BB_ parameters for the individual components11
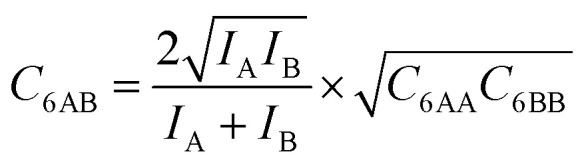
The factor involving the ionization potentials *I* in front of (*C*_6AA_*C*_6BB_)^1/2^ in eqn (11) is close to unity, see the ESI, Section S2,† which also contains a more elaborate derivation of the GM mixing rule from Lenard-Jones potentials.

Our lattice GCMC method offers the unique possibility of examining the AM approximation without any additional assumptions. If in every step of the GCMC simulation, the A⋯B interaction energies are approximated as the AM of A⋯A and B⋯B pair interaction energies, we refer to it as “GCMC-AM”, see Scheme 1. In addition, we will use the analytical and computationally much more efficient competitive MF (CMF) model for testing the AM mixing rule and for comparing it with the GM mixing rule. We will insert *E*^av^_AB_ values in the CMF equations, eqn (8), that are approximated according to eqn (1) and (2). We will refer to the results as “CMF-AM” and “CMF-GM”, respectively, see Scheme 1.

### 3.4. *Ab initio* lattice GCMC and CMF results for co-adsorption isotherms and selectivities

The adsorption selectivity coefficients *s*_A,B_ are defined as the ratio of the mole fractions *x*_c_ of different gas mixture components in the adsorbed phase divided by the respective mole fraction ratios in the gas phase, *y*_c_,12
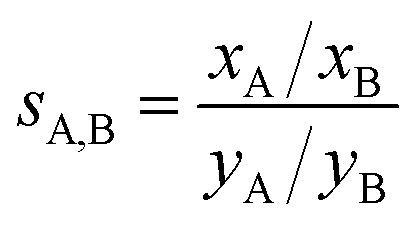


#### Ideal mixtures

For CO/N_2_ and CH_4_/N_2_ mixtures, the similar strengths of the lateral interactions between CO, N_2_ or CH_4_ molecules adsorbed at the Mg^2+^ sites, −0.34, −0.35, and −0.55 kJ mol^−1^, respectively (Table 1), result in constant selectivity values with varying total pressure or composition, see the ESI, Fig. S3.†Table 2 shows that the averages of the *ab initio* mixed lateral interaction energies, *E*^av^_AB_(*ab initio*), differ little from the AM of the pure component lateral interaction energies, *E*^AM^_AB_. Consequently, the energy of mixing, eqn (9) is almost zero and these adsorbed phases behave ideally. Therefore, the selectivities obtained from GCMC-AM simulations are virtually identical with the GCMC selectivities.

The selectivity of 1.1 calculated for a wide range of CH_4_/N_2_ mixture compositions and gas phase pressures shows that it will not be possible to separate nitrogen impurities from natural gas. For CO/N_2_, our *ab initio* lattice GCMC simulations yield a selectivity value of 8.4 which agrees well with the IAST selectivities of around 10 calculated for different CO/N_2_ mixtures based on measured pure gas data.^17^

#### Non-ideal mixtures

**Fig. 2:**
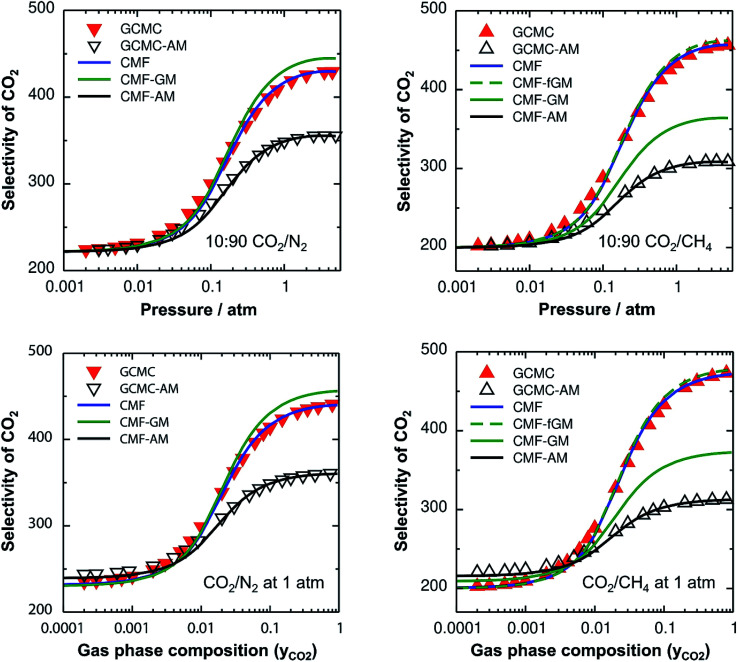
CO_2_ selectivities for CO_2_/N_2_ (left) and CO_2_/CH_4_ mixtures (right) as a function of total pressure (top) for a mixture with 10% CO_2_ and as a function of the gas phase composition (bottom) for a total pressure of 1 atm, both at 298 K. Red inverted triangles and triangles show the GCMC results for CO_2_/N_2_ and CO_2_/CH_4_, respectively, whereas blue lines represent the CMF results. Open triangles and black lines show the GCMC and CMF results, respectively, with the arithmetic mean mixing rule for A⋯B interactions, GCMC-AM and CMF-AM, respectively. Green solid and green broken lines show the CMF results with the geometric mean mixing rule, without and with the anisotropy factor, respectively.

The lateral interactions between adsorbed CO_2_ molecules are much stronger than those between CH_4_ and N_2_, by a factor of 5 and 8, respectively (Table 1). This results in substantial mixing energies for the CO_2_/N_2_ and CO_2_/CH_4_ mixtures (Table 2) and the adsorption selectivities become a function of the total pressure and the gas composition, see Fig. 2. We first discuss the GCMC results which will serve as a reference for all other simulations. For a mixture with 10% CO_2_, the CO_2_ adsorption selectivity does not change up to a pressure of 0.02 atm because the surface coverage is so low that the adsorbed molecules can be regarded as isolated. Upon further increase of pressure, the CO_2_ adsorption selectivity increases by about a factor of 2, reaching, at 2 atm, 425 and 450 units for CO_2_/N_2_ and CO_2_/CH_4_, respectively. Similar increases of the adsorption selectivities occur also for increasing CO_2_ content in the CO_2_/N_2_ and CO_2_/CH_4_ mixtures at a total pressure of 1 atm (Fig. 2 bottom).

#### Comparison of GCMC and CMF selectivities

Fig. 2 shows the comparison of the CO_2_ selectivities obtained with lattice GCMC simulations (“GCMC”, symbols) to those obtained with the competitive MF equations (“CMF”, lines) using the same *ab initio* results for Gibbs free energies of adsorption (Table 1) and lateral interaction energies (Table 2) as the input. From the excellent agreement we conclude that co-adsorption isotherms and selectivities can be calculated analytically using the CMF model without sacrificing accuracy and without the substantial computational cost of GCMC simulations. While the former are obtained at no cost, the latter may require an hour of computer time on a single-core processor for each point of the isotherm.

#### Arithmetic mean approximation

For the ideal CO/N_2_ and CH_4_/N_2_ mixtures, the GCMC selectivities neither depend on the pressure nor on the gas composition. The mixing energies are very small (Table 2) and there is no effect of the AM approximation (ESI, Fig. S3†).

For the non-ideal CO_2_/N_2_ and CO_2_/CH_4_ mixtures, GCMC-AM substantially underestimates the selectivity for CO_2_ – the major component on the surface – by 16% and 32% for 10 : 90 mixtures of CO_2_/N_2_ and CO_2_/CH_4_, respectively, in the pressure range between 0.5 and 5 atm, see Fig. 2, top. Correspondingly, the adsorbed amounts of the minor components, *i.e.*, N_2_ and CH_4_ for CO_2_/N_2_ and CO_2_/CH_4_, respectively, are overestimated with the AM approximation. The reason is that the CO_2_⋯CO_2_ lateral interactions are stronger than the N_2_⋯N_2_ and CH_4_⋯CH_4_ ones, which makes the AM for the CO_2_⋯N_2_ and CO_2_⋯CH_4_ interactions, *E*^AM^_AB_, 46% and 158%, respectively, larger than the explicitly calculated *E*^av^_AB_(*ab initio*) values, see Table 2. Consequently, the stabilities of the minor components on the surface are overestimated which leads to the overestimation of their adsorbed amounts with the AM approximation. The dependence of the selectivity on the gas phase composition (bottom panels of Fig. 2) also shows that the AM approximation underestimates the GCMC CO_2_ selectivity for a high CO_2_ content, by 18% and 34% for CO_2_⋯N_2_ and CO_2_⋯CH_4_, respectively.

The effect of the AM mixing rule seen with lattice GCMC simulations (GCMC-AM in Fig. 2) is reproduced by the CMF model if the AM approximation is applied to calculate the average lateral interactions in eqn (8), CMF-AM. Because of this and because the CMF model is computationally much more efficient, in the following we will use the CMF to test the geometric mean approximation.

#### Geometric mean approximation

Table 2 shows the comparison of the average A⋯B interaction energies, *E*^av^_AB_(*ab initio*), obtained from *ab initio* calculations with the AM and the GM of A⋯A and B⋯B interaction energies. For weakly interacting mixtures, *i.e.*, CH_4_/N_2_ and CO/N_2_, both the AM and GM approximations are comparable with *E*^av^_AB_(*ab initio*). For CO_2_⋯N_2_ interaction energy, if the GM approximation is used, the deviation from *E*^av^_AB_(*ab initio*) is reduced to only 8%, while the deviation was 46% with the AM approximation. For CO_2_⋯CH_4_, the GM is also a significant improvement compared to the AM, 91% compared to 158%, but the remaining deviation is still substantial.

Fig. 2 shows the comparison of the AM and GM approximations for CMF selectivities, CMF-AM and CMF-GM, respectively. For CO_2_/N_2_ mixtures, CO_2_ selectivities obtained with GM are in close agreement with the CMF results. The reason is that the GM of the *E*^av^ parameters of CO_2_ and N_2_, *E*^GM^_AB_ = −1.0 kJ mol^−1^ (see Table 2), is very close to the average of the directly calculated lateral interaction energies between CO_2_ and N_2_ molecules in the adsorbed phase, *E*^av^_AB_(*ab initio*) = −1.1 kJ mol^−1^.

For CO_2_/CH_4_, Fig. 2 shows an improvement of about 13% for the calculated selectivities when GM instead of AM mixing is used, but the deviations from the CMF results are still substantial, about 20%. The reason is that *E*^GM^_AB_ = −1.24 kJ mol^−1^ gets closer to *E*^av^_AB_(*ab initio*) = −0.65 kJ mol^−1^ than *E*^AM^_AB_ = −1.68 kJ mol^−1^, but the deviation is still 0.59 kJ mol^−1^. The origin of the remaining deviations observed with the CMF-GM model is the anisotropy of the lateral interaction energies (*E*_AB_) for “mixed” pairs as our *ab initio* calculations show. This anisotropy will be addressed in the next section.

Co-adsorption selectivity, nevertheless, is independent of this anisotropy if adsorbed molecules do not interact with each other on the surface, *e.g.*, at a very low surface coverage. In this case, the co-adsorption selectivity becomes the ratio of zero coverage equilibrium constants for both components. Using the *ab initio* values for the latter, the calculated zero coverage CO_2_/N_2_ selectivity is 167 at 313 K and the CO_2_/CH_4_ selectivity is 199 at 298 K, which are in good agreement with the experimental IAST selectivities of *ca.* 175 and 210–220, respectively.^52–54^

### 3.5. Anisotropy factor

For the CO_2_/CH_4_ mixture, the geometric mean of the CO_2_⋯CO_2_ and CH_4_⋯CH_4_ interaction energies, *E*^GM^_AB_ = −1.24 kJ mol^−1^, is almost twice as large as the average *ab initio* CO_2_⋯CH_4_ interaction energies, *E*^av^_AB_(*ab initio*) = −0.65 kJ mol^−1^ (Table 2). This can be explained by the “symmetry breaking” seen in Fig. 1 that yields a strongly interacting CO_2_⋯CH_4_ pair at short distance and a weakly interacting pair at long distance. The interaction energy of the former (−1.07 kJ mol^−1^) differs only 0.17 kJ mol^−1^ from the GM (−1.24 kJ mol^−1^) of the individual lateral interaction energies, whereas the interaction energy of the weakly interacting pair (−0.22 kJ mol^−1^) differs 1.02 kJ mol^−1^ and is even less than half of the CH_4_⋯CH_4_ interaction energy (−0.55 kJ mol^−1^).

We may take this into account by defining effective pair interaction energies regarding the weakly interacting CO_2_⋯CH_4_ pairs as non-interacting. Assuming that there are *n*_AA_, *n*_BB_ and *n*_AB_ numbers of A⋯A, B⋯B and A⋯B pairs, respectively, with non-negligible interaction energies we get:13
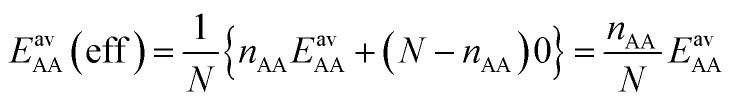
14
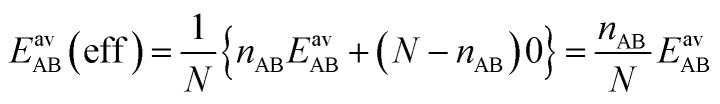


If we apply the geometric mean approximation for the average interaction energy of a mixed pair, *i.e.*,15
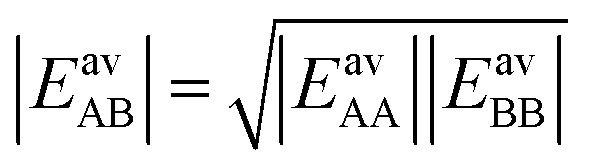
and then substitute the expressions for *E*^av^_AA_ and *E*^av^_AB_ from eqn (13) and (14), respectively, we obtain16
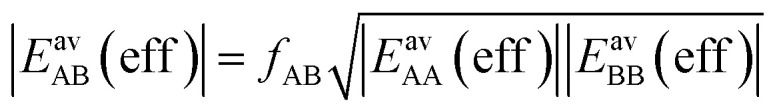
with17
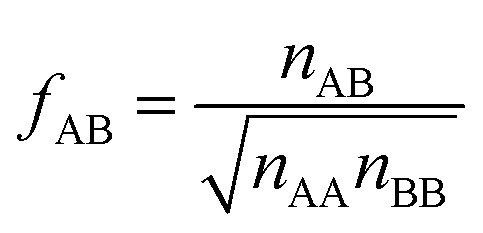
We will call *f*_AB_ the anisotropy factor. If the number of interacting “mixed” pairs is equal to the number of interacting “same” pairs, *i.e.*, *n*_AB_ = *n*_AA_ = *n*_BB_, then *f*_AB_ will be unity and consequently, the simple geometric mean will be the correct combination rule to obtain *E*^av^_AB_(eff). However, if the anisotropy of lateral interactions on the surface makes the number of interacting neighbors different for different pair types, *i.e.*, AA, BB and AB pairs, the anisotropy factor *f*_AB_ will no longer be unity and has to be taken into account for accurate predictions of co-adsorption selectivities,18
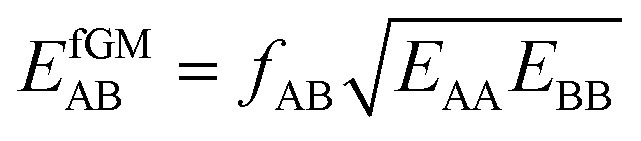
The results obtained with inclusion of the anisotropy factor, eqn (18), are named “CMF-fGM”.

For the CO_2_/CH_4_, the long, weakly interacting (−0.22 kJ mol^−1^) CO_2_⋯CH_4_ pairs are regarded as non-interacting which means that there is only one interacting “mixed” pair, *n*_AB_ = 1, whereas there are two interacting “same” pairs, *n*_AA_ = *n*_BB_ = 2, hence
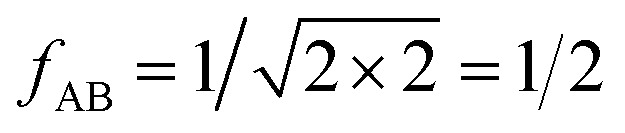


After inclusion of the factor 1/2 for the CO_2_⋯CH_4_ interaction, we get *E*^GM^_AB_/2 = −0.62 kJ mol^−1^ which nicely agrees with the *ab initio* energy for the same interaction, *E*^av^_AB_(*ab initio*) = −0.65 kJ mol^−1^. Moreover, the effective mixing energy, Δ*E*^GM/2^_mix_ = 1.06, calculated using the former, matches well with that for the “real mixture”, *ΔE*_mix_ = 1.04 kJ mol^−1^, see Table 2. As Fig. 2 shows, the CMF calculations that account for the anisotropy factor *f*_AB_ = 1/2, CMF-fGM, reproduce the target CMF and GCMC results.

## 4. Prediction of co-adsorption isotherms and selectivities from pure gas adsorption data

### 4.1. Ideal adsorbed solution theory (IAST)

Ideal adsorption solution theory (IAST)^15^ is the most widely used model for prediction of co-adsorption from pure gas adsorption isotherms.^15,16^ Solving the IAST equations involves integration over isotherms,^15^ see Section S5 of the ESI† for details. According to the standard protocol,^38^ first pure gas data points are fitted with an analytical isotherm model and subsequently the integration over isotherm expressions is done analytically.^38^

For fitting the single component data points, we make use of the non-linear mean-field isotherm equation,^36^19
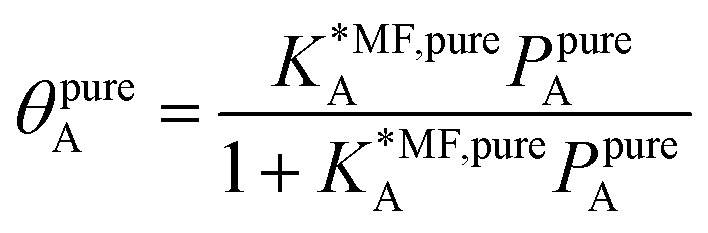
with20
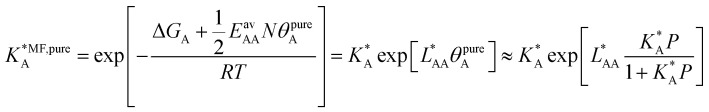
Inside the exponential function the dependency of the mean field equilibrium constant, 
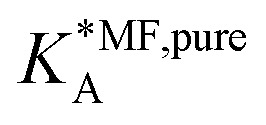
, on the surface coverage is approximated using a Langmuir isotherm. From the fitting parameters 
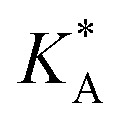
 and 
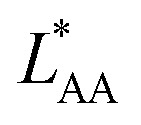
, the Gibbs free energies of adsorption and the average lateral interaction energies are obtained respectively, according to21
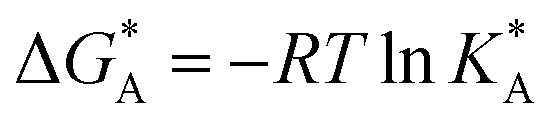
22
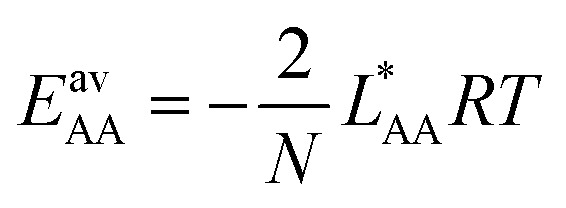


Table 1 shows the comparison of the fitted parameters with the *ab initio* data used for the GCMC simulations. For adsorbed gases with very weak lateral interactions (CO, N_2_, and CH_4_) there is agreement within 0.1 kJ mol^−1^; for CO_2_ with stronger lateral interactions (−2.8 kJ mol^−1^) the fit yields 0.4 kJ mol^−1^ (15%) stronger binding. The agreement can be improved by further expanding 
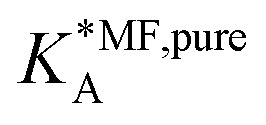
 in continued fraction representation, see the ESI, Section S6.†

IAST predictions for mixture isotherms according to the standard protocol described here will be labelled “IAST” in the following, see Scheme 1. Any kind of pure component isotherms can be used, originating either from experiments^16,38^ or from simulations. The simulation method employed for the pure component isotherms is irrelevant, and it may range from *ab initio* GCMC on a lattice of sites (as we use in this work)^34–36^ to GCMC simulations in full configuration space using a force field, see, *e.g.*, ref. 55.

**Fig. 3:**
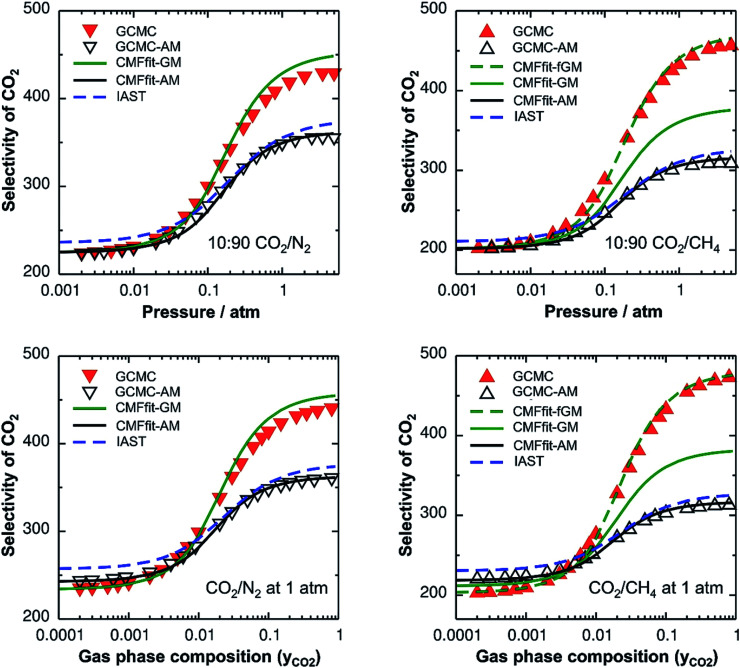
CO_2_ selectivities for CO_2_/N_2_ (left) and CO_2_/CH_4_ (right) mixtures. The top panel shows the variation of selectivities as a function of total pressure for a mixture containing 10% CO_2_, whereas the bottom panel shows the variation of selectivities as a function of gas phase composition. Symbols (inverted triangles: CO_2_/N_2_ and triangles: CO_2_/CH_4_ mixtures) represent the results obtained from the reference GCMC simulations. Red and black symbols are for “GCMC” and “GCMC-AM” simulations, respectively. Lines represent the CMF results obtained with parameters from linear mean field fits of pure gas isotherms for different mixing rules. The blue broken lines represent the IAST calculations.

Since IAST shares the assumption of ideal mixtures with GCMC-AM and CMF-AM (Fig. 3), eqn (1), we should expect to get the same results. This is shown in Fig. 3 which will be discussed in more detail below.

### 4.2. Competitive mean field model with arithmetic mean approximation

The AM combination rule, together with the CMF model, opens another way of predicting co-adsorption isotherms and selectivities from measured or simulated pure component isotherms similar to IAST. Also here, the first step is to fit the isotherm data points with mean-field isotherm models. For a pure gas A, eqn (4) becomes,23
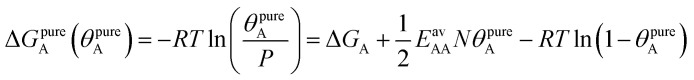
Rearranging the above equation, we get,24
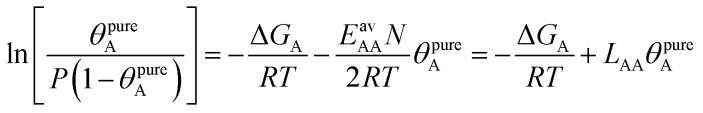
The intercept of the plot ln[*θ*/(*P*(1 − *θ*))] *vs. θ* gives the Gibbs free energy of adsorption at zero coverage divided by −*RT* and the slope is the adsorbate–adsorbate lateral interaction energy parameter, *L*_AA_. Eqn (24) is identical to the linear form^56^ of the Fowler–Guggenheim adsorption isotherm. The data points of the isotherms are then obtained from the Δ*G*_A_ and *L*_AA_ parameters in an iterative procedure according to eqn (19) and (20). This is the reason why this linear fitting approach could not be used with the IAST standard protocol (Section 4.1) which involves an analytical integration.

Table 1 shows that the linear, free energy fitting procedure, eqn (24), reproduces the pure component lateral interaction energies for adsorbed gases with very weak lateral interactions, *i.e.*, for CO, N_2_, and CH_4_, within 0.1 kJ mol^−1^ of the directly calculated *ab initio* values, and within 0.2 kJ mol^−1^ for adsorbed gases with stronger lateral interactions, *e.g.* CO_2_.

In the second step, the fitted *L*_AA_ and *L*_BB_ interaction parameters are used to approximate *L*_AB_ as the arithmetic mean, *cf.*eqn (1),

25 *L*^AM^_AB_ = (*L*_AA_ + *L*_BB_)/2

and the competitive mean field (CMF) model, eqn (6) and (8), is adopted to predict co-adsorption isotherms. We refer to these isotherms as “CMFfit-AM”, see Scheme 1.

With this, we have two different protocols for generating co-adsorption isotherms from pure component ones, both based on the assumption of ideal mixtures, IAST and CMFfit-AM. Since we use the same *ab initio* data as the input, they should yield the same results. While the IAST protocol does not require any explicit specification of the interactions between different adsorbed molecules, but involves an integration step, the CMFfit-AM protocol relies on the AM approximation for the interaction between pairs of unlike adsorbate molecules.

### 4.3. Results: IAST and CMF fit with the arithmetic mean mixing rule

#### Ideal mixtures

For CH_4_/N_2_ and CO/N_2_, as we have already seen from the GCMC-AM results in Fig. S3 in the ESI,† the predicted “IAST” and “CMFfit-AM” selectivities are in perfect agreement with those obtained from the reference “GCMC” simulations – all predict constant selectivity values with varying pressure or composition, see Fig. S4 in the ESI.† This is expected because the mixed pair interaction energies, −*RTL*_AB_, as well as the mixing energies, −*RT*Δ*L*_mix_, are very close to the original *ab initio* values, *E*^av^_AB_(*ab initio*) and Δ*E*_mix_, respectively, see Table 2.

#### Non-ideal mixtures

For CO_2_/N_2_ and CO_2_/CH_4_ mixtures, Fig. 3 shows that the selectivities obtained with the standard “IAST” protocol and the “CMFfit-AM” co-adsorption model are in good agreement with each other. When the CO_2_ selectivities obtained in these two ways are compared with the “GCMC-AM” simulations, there is also good agreement, only for mixtures with a low CO_2_ content, *i.e.* in the low surface coverage region, IAST calculations overestimate the CO_2_ selectivities, while the CMFfit-AM results are still in good agreement with the GCMC-AM results. This is due to the fact that for fitting of pure gas isotherms with a non-linear MF model (as done for IAST), data points up to a very high coverage (90%) are used and the obtained fitting parameters may not represent the low surface coverage region so well.

### 4.4. Competitive mean field model with geometric mean approximation

In our CMF calculations with *ab initio* data (Section 3.4) the GM approximation for mixed terms proved to be superior to the AM approximation. This also suggests improved results when the CMF equations, eqn (6), are used with the GM of the fitted *L*_AA_ and *L*_BB_ interaction parameters,26
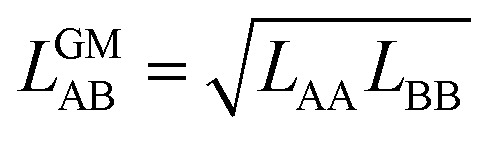
We refer to these isotherms as “CMFfit-GM”, see Scheme 1.

The anisotropy factor *f*_AB_, eqn (17), that has further improved the CMF-GM results for CO_2_/CH_4_ cannot be derived from pure gas adsorption data, neither from experimental nor from simulated ones. It requires input from adsorption structures for the mixture. The latter is only available from atomistic simulations, but high-level quantum chemical calculations as we perform in this study are not required. Computational methods that are easily available also for non-specialists like DFT(+dispersion) or even simple force fields, *e.g.*, ref. 17, 24, and 25 are sufficient as long as they provide qualitatively correct adsorption structures. We will refer to such methods as “computational” in the following.

After identifying the location of the adsorption sites, one needs to carry out three DFT (or force field) optimization runs for the MOF structures with all adsorption sites filled with: (i) A, (ii) B, and (iii) half A and half B. In structure (iii), A and B molecules should alternate at the adsorption sites (see Fig. 1). Then lateral interaction energies for each “same” pair (AA, BB) and “mixed” (AB) pair must be calculated explicitly.

In Section 3.5 the numbers of interacting neighbors were determined by inspection of the structure of the adsorbate layer for the mixture and from these numbers, *f*_AB_ was calculated using eqn (17). For a generally applicable protocol we recommend a different approach that is easier to implement into a computer code. Here, the anisotropy factor is calculated directly from the computed average lateral interaction energies. The GM approximation27
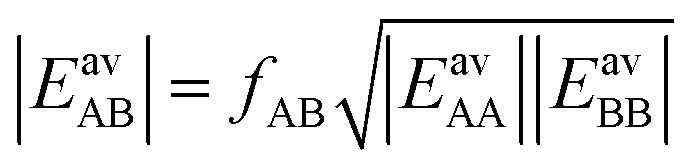
becomes exact if28
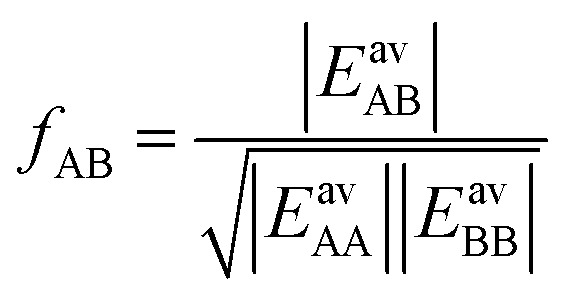


Not much is gained so far, because we need to know *E*^av^_AB_ to calculate *f*_AB_. However, we may calculate *f*_AB_ from the results of a simple computational approach mentioned above,29
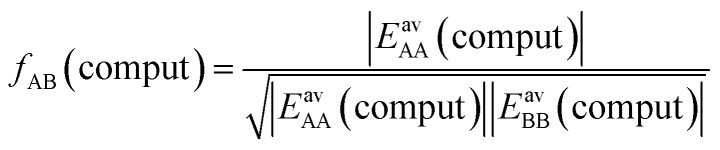
and use the fitted *L*_AA_ and *L*_BB_ interaction parameters to approximate *L*_AB_ within the competitive mean field model as30
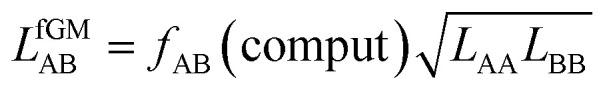
We refer to co-adsorption isotherms obtained this way as “CMFfit-fGM”, see Scheme 1.

The *f*_AB_ values calculated according to eqn (29) for CO_2_/N_2_ and CO_2_/CH_4_ are 1.09 and 0.52, respectively. The latter is in agreement with *f*_AB_ = 0.5 obtained in Section 3.5 for CO_2_/CH_4_ from the numbers of non-interacting pairs. For the isotherms reported in Fig. 2 and 3 for CO_2_/CH_4_, we used *f*_AB_ = 0.5.

Table 2 shows the GM mixed pair interaction energies, −*RTL*^GM^_AB_, as well as the GM mixing energies, −*RT*Δ*L*^GM^_mix_. They have been obtained from single component *L*_AA_ and *L*_BB_ parameters that resulted from linear MF fitting of the GCMC isotherms for pure components. Table 2 shows that they are very close to the directly calculated *E*^GM^_AB_ and Δ*E*^GM^_mix_ values. We therefore expect that our “CMFfit-GM” and “CMFfit-fGM” co-adsorption isotherms show the same improvements over “CMFfit-AM” (Fig. 3) as Fig. 2 shows for the “CMF-GM” and CMF-fGM” compared to CMF-AM” – and this is indeed the case.

For CO_2_/N_2_ mixtures the “CMFfit-GM” predicted selectivities deviate only 4% from the GCMC reference values. This excellent agreement is reached because the GM of the *L*-parameters for CO_2_ and N_2_ (−0.9 kJ mol^−1^) is very close to the average *ab initio* lateral interaction energy, *E*^av^_AB_(*ab initio*) = −1.1 kJ mol^−1^, for a CO_2_⋯N_2_ pair, see Table 2. Moreover, the mixing energy calculated using these *L*-parameters is also within 0.1 kJ mol^−1^ of the *ab initio* mixing energy, Δ*E*_mix_.

For CO_2_/CH_4_, CMFfit-GM also improves the CO_2_ selectivities compared to CMFfit-AM, though the deviations from the reference GCMC results can be as large as 20%. The reason is that the geometric mean (−1.1 kJ mol^−1^) improves −*RTL*_AB_ by 0.5 kJ mol^−1^ compared to the arithmetic mean (−1.6 kJ mol^−1^), yet it is almost double the *ab initio* CO_2_⋯CH_4_ lateral interaction energy, *E*^av^_AB_(*ab initio*) = −0.65 kJ mol^−1^. After including an anisotropy factor of 0.5, the −*RTL*_AB_-parameter (−0.56 kJ mol^−1^) is within 0.1 kJ mol^−1^ of the *E*^av^_AB_(*ab initio*) for CO_2_⋯CH_4_ interaction, and the mixing energy calculated using these *L*-parameters (−*RT*Δ*L*_mix_) is in perfect agreement with the *ab initio* energy of mixing, Δ*E*_mix_. Fig. 3 shows that for CO_2_/CH_4_ the CMFfit-fGM results deviate by less than 1% from the GCMC reference values.

### 4.5. Co-adsorption mean field theory (CAMT)

For adsorbents with distinct adsorption sites and relatively weak lateral adsorbate–adsorbate interactions compared to adsorbate surface site interactions, we have shown that predictions of co-adsorption isotherms and adsorption selectivities from pure gas isotherms always improve when the GM instead of the AM mixing rule is applied, regardless whether the anisotropy factor is introduced or not. We have introduced GM mixing based on the London formula for dispersion, but intermolecular perturbation theory in combination with intermolecular multipole expansion tells us that also induction and electrostatic interaction terms contain products of multipole moments or multipole polarizabilities of the individual components. We therefore expect that the GM mixing will always be an improvement compared to the AM mixing assumed (implicitly) by IAST, even when applied to systems with stronger adsorbate–adsorbate interaction, for example hydrogen bonded water/alcohol, alcohol/alcohol or alcohol/aromatic mixtures in MOFs and zeolites.^57^

We therefore recommend our computational protocol based on the GM mixing rule for general use when predicting co-adsorption isotherms from pure gas data. As IAST, our method which we name co-adsorption mean field theory (CAMT) needs fitted pure gas isotherms only, either from experiments or from simulation. The difference is that CAMT needs fitting with the mean field form that yields parameters for lateral interactions. CAMT involves the following steps:

(i) Mean field fitting of pure gas adsorption data, eqn (24), yielding Gibbs free energies for gas–surface interactions and gas–gas (A⋯A and B⋯B) interaction energies;

(ii) Competitive mean field calculation, eqn (6) and (8), of co-adsorption isotherms using pure gas data and A⋯B interaction energies approximated as the geometric mean of the single component A⋯A and B⋯B interaction energies, eqn (26).

Further improvement can be expected if a third, optional step is made which, however, requires input from computational methods:

(iii) Performing three structural optimizations using force fields or DFT for the adsorbent loaded both with the pure gases and the mixture, and calculation of the anisotropy factor in eqn (30) according to eqn (29). This does not necessarily require CCSD(T) calculations for the isolated pair interactions *E*^av^ (comput) at the periodic structures. As Table S4 in the ESI† shows, DFT-D calculations for the isolated pairs yield very similar values for *f*_AB_.

## 5. Summary and conclusions

We have used GCMC simulations on a lattice of Mg^2+^ adsorption sites with *ab initio* data as the input (eqn (3)) to predict co-adsorption isotherms and selectivities for CH_4_/N_2_, CO/N_2_, CO_2_/N_2_ and CO_2_/CH_4_ mixtures interacting with the Mg^2+^ ions of the Mg_2_(dobdc) MOF. We have also presented competitive mean field (CMF) equations that use average values for lateral interaction energies instead of calculating the lateral interactions for every single adsorbate configuration explicitly as GCMC simulations do. With the same *ab initio* data as the input, we have found excellent agreement between the predictions of the two methods and conclude that the computationally much more efficient CMF equations can be safely used to predict mixture isotherms.

With both the GCMC and CMF methods we have tested the arithmetic mean (AM) of A⋯A and B⋯B adsorbate–adsorbate interactions as approximation for A⋯B interactions and obtained the same results, which also agreed with the standard IAST protocol for predicting co-adsorption isotherms from pure component isotherms. For the CH_4_/N_2_ and CO/N_2_ mixtures in which both gases have very weak lateral interactions, use of AM mixing or IAST shows very good agreement with the exact GCMC and CMF predictions, whereas for mixtures which contain the more strongly interacting CO_2_ (CO_2_/N_2_ and CO_2_/CH_4_) substantial deviations from ideal behavior are found.

If GM mixing is applied instead of AM mixing, as the laws of intermolecular interactions such as the London formula for dispersion suggest, agreement with the exact CMF (and GCMC) results is perfect for CO_2_/N_2_. For CO_2_/CH_4_ substantial improvement is reached, and the remaining deviation can be explained by the anisotropy of the lateral interactions in the mixed adsorbate layer. After introducing a factor (1/2 in this case) that accounts for this anisotropy, very good agreement is also reached for CO_2_/CH_4_.

For predicting co-adsorption isotherms and selectivities from pure gas data, we suggest a new computational protocol, co-adsorption mean field theory (CAMT) that like IAST starts from fitting isotherm expressions to pure gas adsorption data, but unlike IAST uses mean field theory for fitting and applies the GM mixing rule to approximate the interaction energies between different components in the CMF equations for the mixture.

Because of the generality of the GM for parameters describing intermolecular interactions, we expect an improvement compared to IAST in all cases in which adsorbate–adsorbate interactions are smaller than adsorbate–surface interactions. The anisotropy factor cannot be derived from pure gas data only; it requires atomistic structural optimizations for the pure gas and mixed adsorbate layers, using either force fields or DFT.

The present work has considered binary mixtures on homogeneous surfaces with identical surface sites. Future studies should aim at incorporating surface heterogeneity, for example linker sites for MOFs.

## Conflicts of interest

There are no conflicts to declare.

## Supplementary Material

SC-011-C9SC03008E-s001
