# Supplementary material for: Predicting adsorption selectivities from pure gas isotherms for gas mixtures in metal–organic frameworks
Source: Chem Sci. 2019 Dec 6;11(3):643–55. doi: 10.1039/c9sc03008e (PMC8146500; doi:10.1039/c9sc03008e)
Supplement: SC-011-C9SC03008E-s001 [file SC-011-C9SC03008E-s001.pdf]

Electronic Supplementary Material (ESI) for Chemical Science.
This journal is © The Royal Society of Chemistry 2019

Electronic Supplementary Information

Predicting adsorption selectivities from pure gas isotherms for gas mixtures in metal-organic frameworks

Arpan Kundu,[1, §] Kaido Sillar, [1,2, §] Joachim Sauer* [1]

[1] Humboldt Universität zu Berlin, Institut für Chemie, Unter den Linden 6, 10099 Berlin

[2] University of Tartu, Institute of Chemistry, Ravila 14a, 50411, Tartu, Estonia

[§] equal contribution

S1. Mean Field approximation for lateral interactions in a multicomponent mixture

S2. Derivation of the geometric mean mixing rule

S3. Additional figures for ideal mixtures - CO/N_2 and CH_4/N_2

S4. Lateral interactions and anisotropy factor

S5. Ideal adsorption solution theory (IAST)

S6. Expansion of mean field equilibrium constant for pure gas in continued fraction representation

S7. References

S1. Mean Field approximation for lateral interactions in a multicomponent mixture.

Let us consider a gas mixture with m number of components and let c, c' denote the indices of the gas components. At equilibrium, the Gibbs free energy of adsorption for c-th component of the gas mixture for a given coverage (θ_c) can be calculated from the partial pressure, P_c of that component:

$$\Delta G_c\left(\theta_1,...,\theta_c,...,\theta_m\right) = -RT\ln\left[\frac{\theta_c}{P_c}\right] \tag{S1}$$

For a m-component gas mixture, the coverage dependent Gibbs free energy of adsorption for the component c, under the mean-field approximation can be generalized as[1],

$$\Delta G_c\left(\theta_1,\theta_2,...,\theta_m\right) = \Delta G_c + \frac{1}{2}E_{cc}^{\mathrm{av}}N_{cc}\theta_c + \frac{1}{2}\sum_{c'\neq c}^{m}E_{cc'}^{\mathrm{av}}N_{cc'}\theta_{c'} - RT\ln\left(1-\theta\right);\ c,c' = 1,2,...,m \tag{S2}$$

with $N_{cc'}$ denoting the maximum number of interacting $c\cdots c'$ neighbors. The multiplier ½ avoids the double counting of the interactions. $E_{cc'}^{\mathrm{av}}$ denotes the average lateral interaction energy per pair of adsorbed molecules of c-th and c'-th component of the gas mixture.

$$E_{cc'}^{\mathrm{av}} = \frac{1}{N_{cc'}}\sum_{pair}E_{cc'}(pair) \tag{S3}$$

Here, $E_{cc'}\left(pair\right)$ is the ab initio interaction energy for a particular pair of adsorbed molecules of components c and c', respectively.

The first term on the right-hand side of the Eq. (S2), ΔG_c is the coverage independent Gibbs adsorption free energy and is related to the zero coverage adsorption equilibrium constant, $K_c = \exp\left[-\Delta G_c/RT\right]$. ΔG_c is the binding strength of gas component c to the isolated adsorption sites. The second and third terms of the right-hand side of Eq. (S2) are the lateral interaction energies between the gas molecules of same (cc, pure gas term) and different components (cc', $c' \neq c$, mixing term), respectively. The last term is the configurational entropy (Langmuir entropy term). Here, it is assumed that molecules of different components are distributed randomly on the surface, which was also previously assumed by Fowler and Guggenheim[2]. This assumption makes the configurational entropy term, i.e., the last term in

Eq. (S2), dependent only on the total coverage, which is the sum of coverages of all components, i.e., $\theta = \sum_c^m \theta_c$.

Comparing the right-hand sides of the Eq. (S1) and (S2), we get,

$$-RT\ln\left[\frac{\theta_c}{P_c}\right] = \Delta G_c + \frac{1}{2}E_{cc}^{\text{av}}N_{cc}\theta_c + \frac{1}{2}\sum_{c'\neq c}^{m}E_{cc'}^{\text{av}}N_{cc'}\theta_{c'} - RT\ln(1-\theta) \tag{S4}$$

The mean-field equilibrium constant for the c-th mixture component is,

$$K_c^{\text{MF,mix}}(\theta_1,\theta_2,\ldots,\theta_m) = \exp\left[-\left(\Delta G_c + E_{cc}^{\text{av}}N_{cc}\theta_c/2 + \sum_{c'\neq c}^{m}N_{cc'}E_{cc'}^{\text{av}}\theta_{c'}/2\right)\Big/RT\right]$$

$$\Delta G_c + \frac{1}{2}E_{cc}^{\text{av}}N_{cc}\theta_c + \frac{1}{2}\sum_{c'\neq c}^{m}E_{cc'}^{\text{av}}N_{cc'}\theta_{c'} = -RT\ln K_c^{\text{MF,mix}} \tag{S5}$$

Replacing the first three terms of the right-hand side of Eq. (S4) using the expression from Eq. (S5) we obtain,

$$-RT\ln\left[\frac{\theta_c}{P_c}\right] = -RT\ln K_c^{\text{MF,mix}} - RT\ln(1-\theta)$$

$$-RT\ln\left[\frac{\theta_c}{K_c^{\text{MF,mix}}P_c}\right] = -RT\ln(1-\theta)$$

$$\frac{\theta_c}{K_c^{\text{MF,mix}}P_c} = 1-\theta$$

$$\theta_c = (1-\theta)K_c^{\text{MF,mix}}P_c \tag{S6}$$

The Eq. (S6) is a system of m equations. Summing over all components on both side of the equation we obtain,

$$\theta = \sum_c^m \theta_c = (1-\theta)\sum_c^m K_c^{\text{MF,mix}}P_c$$

$$\theta = \frac{\sum_c^m K_c^{\text{MF,mix}}P_c}{1+\sum_c^m K_c^{\text{MF,mix}}P_c}$$

$$1-\theta = \frac{1}{1+\sum_{c'}^m K_{c'}^{\text{MF,mix}}P_{c'}} \tag{S7}$$

Inserting this expression of (1-θ) in Eq. (S6), we obtain the simplified version of the competitive mean-field (CMF) model,

$$\theta_c = \frac{K_c^{\text{MF,mix}} P_c}{1 + \sum_{c'}^{m} K_{c'}^{\text{MF,mix}} P_{c'}} \tag{S8}$$

When lateral interactions are non-negligible, $K_c^{\text{MF,mix}}$ becomes a function of coverages and consequently, Eq (S8) turns into a self-consistent equation where the coverage of each gas component, $\theta_c = f\left(1,...,\theta_c,...,\theta_m\right)$, is also a function of itself. We solved this "competitive MF model", using an iterative process with an initial guess of coverages from competitive Langmuir model, i.e., $E^{\text{av}} = 0$. Usually, a few iterations are sufficient to yield converged surface coverages, which is comparable to the GCMC coverages; see left panel of Figure S1 for the results for a 10:90 CO_2/N_2 mixture. Figure S1, right panel, shows that selectivities of CO_2/N_2 are almost converged after the first iteration.

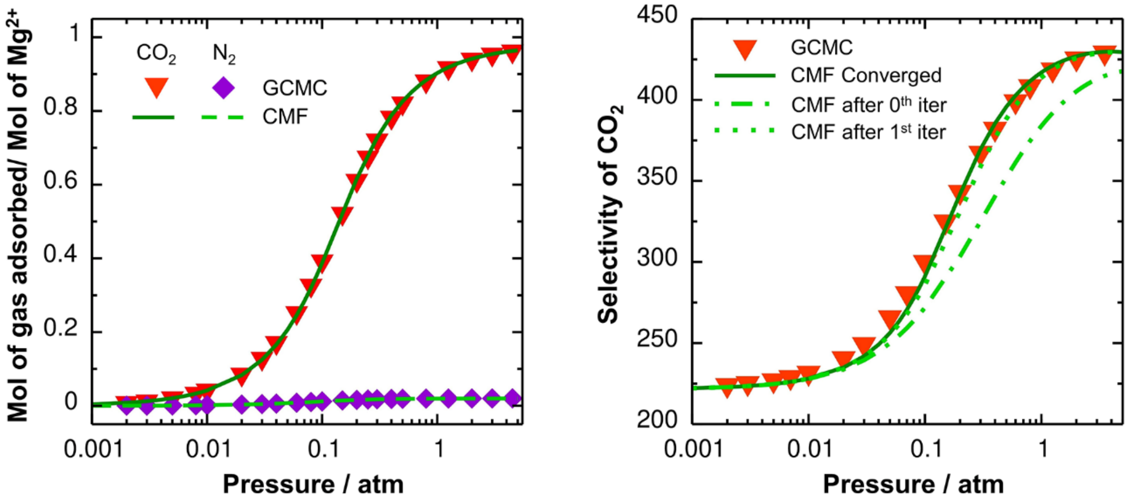

Figure S1. Left: Comparison of CMF converged coverages and GCMC coverages for a 10:90 CO_2/N_2 mixture. Right: Convergence of CMF CO_2 selectivities for a 10:90 CO_2/N_2 mixture with respect to the number of iterations. The convergence threshold for MF calculations were 10^{-6}.

The ab initio CMF calculations are done in two ways: (i) without correcting for the gas phase non-ideality and (ii) correcting for the gas phase non-ideality. In the former just partial pressures for both components are used in Eq (S8), while in the latter calculations partial

pressures are replaced with the respective fugacities. The fugacities for each mixture component are calculated using the Redlich-Kwong equation of state,[3] which is also used in GCMC simulations to account for the gas phase non-ideality.[4]

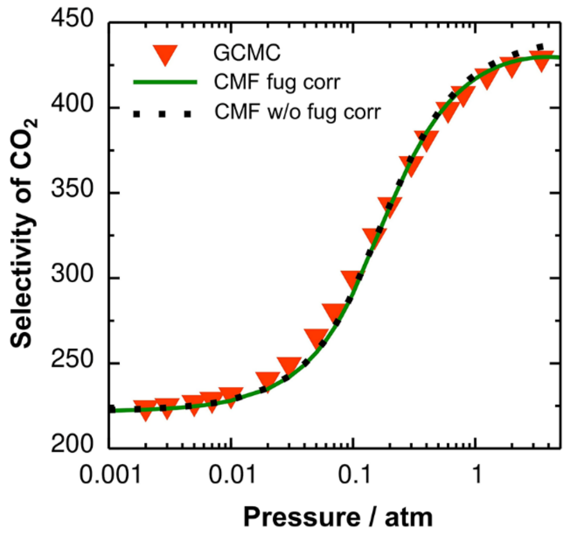

Figure S2. Comparison of CO_2 selectivities obtained from ab initio lattice GCMC (triangles) and ab initio MF (lines) calculations, respectively, for a 10:90 CO_2/N_2 mixture at 298 K.

In Figure S2, the CO_2 selectivities obtained from converged ab initio CMF calculations (lines) are compared with converged ab initio GCMC simulations (red triangles). CMF results, regardless of fugacity corrected or not corrected, are in excellent agreement with the reference GCMC results. A small deviation between the selectivities obtained from MF model without fugacity correction and GCMC simulation is noticeable when total pressure is higher than 2 atm. The reason is that, Redlich-Kwong equation of state[3] predicts a smaller fugacity coefficient for CO_2 than for N_2. Moreover, at pressures higher than 2 atm, the ratio of Redlich-Kwong fugacity coefficients for CO_2 and N_2 decreases with increasing pressure, making CO_2 selectivities almost constant with increasing pressure.

S2. Derivation of the geometric mean mixing rule

The Lennard-Jones potential between two molecules: A and B can be expressed as

$$U_{\mathrm{LJ}}(R_{\mathrm{AB}}) = -4\varepsilon_{\mathrm{AB}}\left[\left(\frac{\sigma_{\mathrm{AB}}}{R_{\mathrm{AB}}}\right)^{6} - \left(\frac{\sigma_{\mathrm{AB}}}{R_{\mathrm{AB}}}\right)^{12}\right] \tag{S9}$$

with R_{AB} and σ_{AB} denoting the center of mass distance and collision diameter (i.e. distance when the inter-atomic potential is zero) between the molecule A and B , respectively. The depth of the potential well, $\varepsilon_{\mathrm{AB}}$, is the interaction energy of the pair of the molecules at the potential-minimum, which appears at inter-molecular distance, $2^{1/6}\ \sigma_{\mathrm{AB}}$. In molecular simulations, the σ and ε parameters for AB pairs are routinely estimated from the average of those parameters for the AA and BB pairs. The collision diameter for the AB pair is chosen as the arithmetic mean of the collision diameters of the AA and BB pairs.[5]

$$\sigma_{\mathrm{AB}} = \frac{1}{2}\left(\sigma_{\mathrm{AA}} + \sigma_{\mathrm{BB}}\right) \tag{S10}$$

This rule, which is known as Lorentz combination rule, is mathematically correct for hard sphere molecules.

The fundamental origin of the attractive part of the Lennard-Jones potential is the London dispersion interaction.[5]

$$U_{\mathrm{London}}\left(R_{\mathrm{AB}}\right) = -\frac{C_{6\mathrm{AB}}}{R_{\mathrm{AB}}^{6}} \tag{S11}$$

Comparing the coefficients of $1/R_{\mathrm{AB}}^{6}$ in Eqs. (S9) and (S11) and utilizing the well-known expression of $C_{6\mathrm{AB}}$ from London approximation we get,

$$4\varepsilon_{\mathrm{AB}}\sigma_{\mathrm{AB}}^{6} = C_{6\mathrm{AB}} = \frac{3}{2}\times\frac{\alpha_{\mathrm{A}}\alpha_{\mathrm{B}}I_{\mathrm{A}}I_{\mathrm{B}}}{I_{\mathrm{A}} + I_{\mathrm{B}}} \tag{S12}$$

where α and I represent the polarizabilities and the ionization potentials of the molecules, respectively. By eliminating α from the above equation, Hudson and McCoubrey obtained the following relation,[6]

$$\varepsilon_{AB} = \varepsilon_{BA} = \frac{\sigma_A^3 \sigma_B^3}{\sigma_{AB}^6} \times \frac{2\sqrt{I_A I_B}}{I_A + I_B} \times \sqrt{\varepsilon_A \varepsilon_B} = \frac{\sigma_A^3 \sigma_B^3}{\left(\frac{\sigma_A + \sigma_B}{2}\right)^6} \times \frac{2\sqrt{I_A I_B}}{I_A + I_B} \times \sqrt{\varepsilon_A \varepsilon_B} \quad \text{(S13)}$$

where Lorentz combination rule, Eq.(S10), is used to approximate the collision diameter, σ_{AB}, for unlike gas molecules.

The b-parameter of the van der Waal's equation of state,

$$\left(P + \frac{a}{\overline{V}^2}\right)\left(\overline{V} - b\right) = RT \quad \text{(S14)}$$

with $\overline{V}$ denoting the molar volume of the gas, can be obtained from critical temperature and pressure measurements of the considered gases and they are available in the literature[7]. If molecules are approximated as hard spheres then it can be shown that the b-parameters are proportional to the volume of a single molecule (V_A),[8]

$$b_A = 4N_{Avo} \times V_A = 4 \times N_{Avo} \times \frac{4}{3}\pi\left(\frac{\sigma_A}{2}\right)^3 \quad \text{(S15)}$$

with N_{Avo} denoting the Avogadro's number. Inserting this result into Eq. (S13) we obtain,

$$\varepsilon_{AB} = \frac{b_A b_B}{\left(\frac{b_A^{1/3} + b_B^{1/3}}{2}\right)^6} \times \frac{2\sqrt{I_A I_B}}{I_A + I_B} \times \sqrt{\varepsilon_A \varepsilon_B} = \tilde{b}_{AB} \times \tilde{I}_{AB} \times \sqrt{\varepsilon_A \varepsilon_B} \quad \text{(S16)}$$

where the $\tilde{b}_{AB}$ and $\tilde{I}_{AB}$ are the correction factors due to different size and different ionization energies of the considered molecules. They have the following expressions,

$$\tilde{b}_{AB} = \frac{b_A b_B}{\left(\frac{b_A^{1/3} + b_B^{1/3}}{2}\right)^6} \quad \text{and} \quad \tilde{I}_{AB} = \frac{2\sqrt{I_A I_B}}{I_A + I_B} \quad \text{(S17)}$$

which can be calculated by using the available experimental data for the van der Waal's b-parameters[7] and the ionization potentials (I)[9] of the considered gas molecules.

Table S1 shows the measured values of van der Waal's a and b parameters together with the ionization energies (I) of the gas molecules considered here. We used this data to calculate the correction factors $\tilde{b}$ and $\tilde{I}$ for the gas mixtures, which are shown in table S2.

Comparison of the van der Waal's b parameters for the different gases confirms the size of the molecules are very similar and consequently the correction factors $\tilde{b}$ are close to unity. All the molecules considered here have also very similar ionization energies as all of their highest occupied molecular orbitals (HOMO) are originating from $2p$-levels. Consequently, the factors $\tilde{I}$ are also close to unity.

Table S1. The measured van der Waal's a and b-parameter, volume of a gas molecules (V_m) and the ionization energies (I) for the gas molecules studied in this work. The numbers in parentheses show the percentage values relative to CO_2.

Gas (A/B)	a (L^2bar/mol^2) [a]	b (L/mol) [a]	$V_c \times 10^{26}$ (L) [b]	I (eV) [c]
CO	1.472 (40.2)	0.03948 (92.1)	1.6387 (92.1)	14.014 (101.7)
N_2	1.370 (37.5)	0.0387 (90.3)	1.6063 (90.3)	15.581 (113.1)
CH_4	2.300 (62.9)	0.04301 (100.3)	1.7852 (100.3)	12.61 (91.5)
CO_2	3.658 (100.0)	0.04286 (100.0)	1.7790 (100)	13.777 (100.0)

[a] From ref.[7] [b] calculated from b using Eq. (S15) [c] From ref[9]

Table S2. Comparison of the correction factors $\tilde{b}$ and $\tilde{I}$ calculated using Eq. (S17) for the gas-mixtures studied in this work. Both correction factors are unity for a pair of same molecules.

Pair (A···B)	$\tilde{b}$	$\tilde{I}$
$CH_4 \cdots N_2$	0.9991	0.9944
$CO \cdots N_2$	0.9999	0.9985
$CO_2 \cdots N_2$	0.9991	0.9981
$CO_2 \cdots CH_4$	0.9999	0.9990

If both of these correction factors are unity, Berthelot's combination rule

$$\varepsilon_{AB} = \sqrt{\varepsilon_{AA}\varepsilon_{BB}} \quad \text{(S18)}$$

is recovered.

S3. Additional figures for ideal mixtures - CO/N_2 and CH_4/N_2

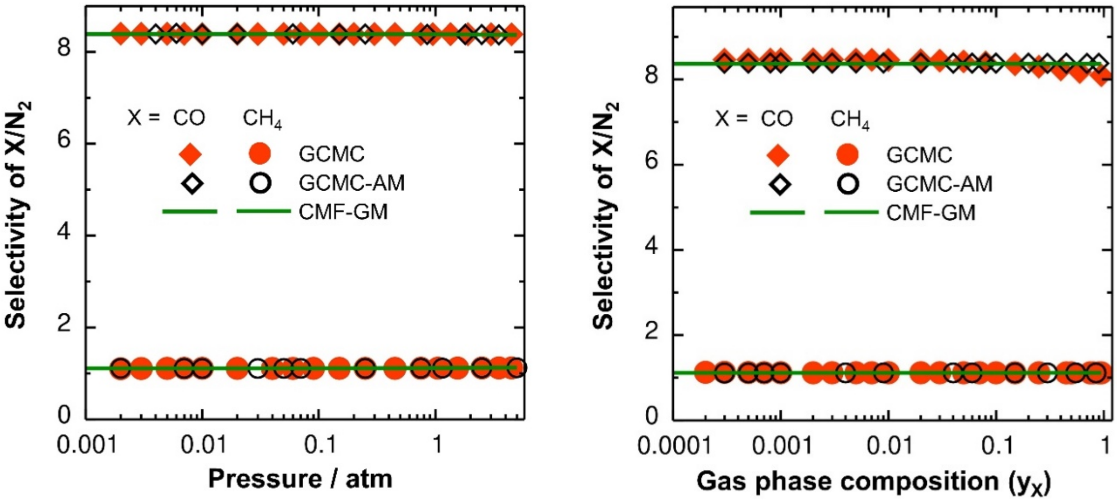

Figure S3. Comparison of selectivities for ideal mixtures obtained from: (i) GCMC simulations which is the target data and (ii) CMF calculations based on ab initio energies. Closed and open symbols (diamonds: CO/N_2, circles: CH_4/N_2) are the selectivities obtained from GCMC and GCMC-AM simulations, respectively. The green lines represent the CMF-GM results. CMF-AM result is identical with GCMC-AM as well as CMF-GM, but not shown for the sake of clarity of the figure. For the nomenclature see scheme-1 in the main text. Fugacity corrections due to gas phase non-ideality are taken into account in all calculations. Left: selectivities of CO(10%) and CH_4(90%) over N_2 as a function of total pressure. Right: CO and CH_4 selectivities as a function of gas phase composition.

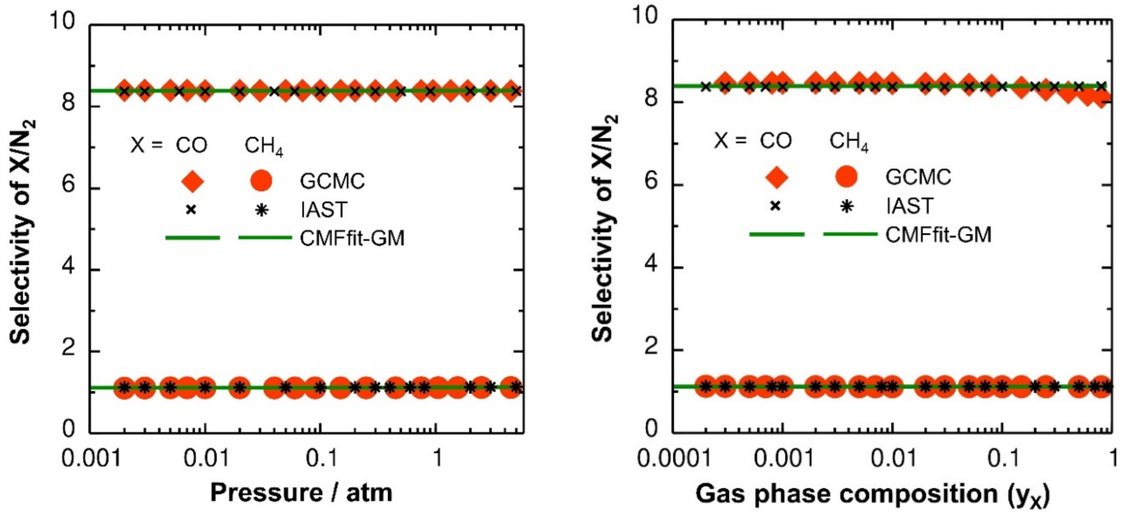

Figure S4. Comparison of selectivities for ideal mixtures obtained from: (i) GCMC simulations which is the target data, (ii) IAST calculations, and (iii) CMFfit-GM calculations based on parameters obtained from linear MF fitting of pure gas isotherms. Symbols represents GCMC selectivities (diamonds: CO/N_2, circles: CH_4/N_2) or IAST calculations (cross: CO/N_2, star: CH_4/N_2). The green lines represent the results obtained from the CMFfit-GM calculations. If CMFfit-AM results are identical with CMFfit-GM as well as IAST, but not shown for the sake of clarity of the figure. See scheme-1 in the main text for the nomenclature. Left: selectivities of CO(10%) and CH_4(90%) over N_2 as a function of total pressure. Right: CO and CH_4 selectivities as a function of gas phase composition.

S4. Lateral interactions and anisotropy factor

Table S3 shows lateral interaction energies calculated for two different models: for the full periodic structures ("In MOF") and the isolated pairs of adsorbed molecules cut from these periodic structures ("Isolated"). For comparison of these two models we have approximated the periodic structures CCSD(T) results with the *hybrid MP2:DFT+D* + *ΔCCSD(T)* method as applied before for calculation of adsorption energies.[1, 10, 11] For that finite size model systems (see Figure S5) are cut from the periodic structures and the BSSE corrected lateral interaction energies are calculated by MP2 and PBE+D2 employing the def2-QZVP basis set. The final estimate for the lateral interaction energies in the MOF include higher order correlation effects, *ΔCCSD(T)*, that are estimated as ΔCCSD(T)/CBS = CCSD(T)/CBS − MP2/QZVP for isolated pair of adsorbed molecules and added to the MP2:PBE+D adsorbate-adsorbate energies. Details of the calculations are the same as used in our previous works. [1, 10, 11]

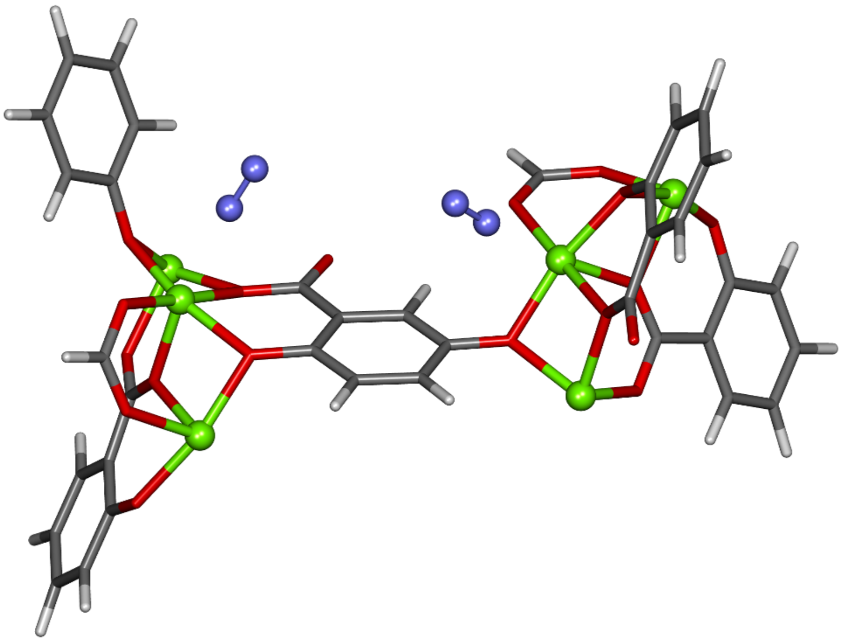

Figure S5. Example of the Model system adopted for the hybrid calculations in case of adsorption pure N_2.

The last column in Table S3, |Hybrid –Isolated CCSD(T)|, shows the lateral interaction energy differences between isolated pair of molecules (in the gas phase) and the same pair in the MOF. The average of these energies is 0.12 kJ/mol. Also, the average lateral interaction energies of the short and long mixed pairs match very well (the average absolute difference is 0.11 kJ/mol) with the geometric mean of the interaction energies of the corresponding pure gas molecules (the "GM – S, L av." rows in Table S3).

Table S3. Lateral interaction energies between pairs of adsorbed molecules calculated with and without the presence of the MOF framework, denoted as In MOF and Isolated, respectively, and lateral interaction energies calculated for mixed pairs from geometric (GM), arithmetic means (AM) of the pure gas data. All in kJ/mol.

	In MOF PBE+D pw	Isolated PBE+D QZVP	Hybrid+ CCSD(T) CBS[a]	Isolated CCSD(T) CBS	|Hybrid - Isolated CCSD(T)|
CO···CO	-0.56	-0.40	-0.51	-0.34	0.17
$N_2 \cdots N_2$	-0.29	-0.40	-0.30	-0.35	0.05
$CH_4 \cdots CH_4$	-0.44	-0.68	-0.49	-0.55	0.06
$CO_2 \cdots CO_2$	-2.36	-2.82	-2.65	-2.81	0.16
**CH_4/N_2**					
Short	-0.47	-0.67	-0.50	-0.54	0.04
Long	-0.29	-0.45	-0.33	-0.40	0.07
S, L av.	-0.38	-0.56	-0.42	-0.47	0.05
GM	-0.36	-0.52	-0.39	-0.44	0.05
AM	-0.37	-0.54	-0.40	-0.45	0.05
GM – S, L av.	0.02	0.04	0.03	0.03	
**CO/N_2**					
Short	-0.92	-0.98	-0.81	-0.81	0.00
Long	-0.16	-0.13	-0.12	-0.14	0.02
S, L av.	-0.54	-0.56	-0.46	-0.48	0.02
GM	-0.40	-0.40	-0.39	-0.34	0.05
AM	-0.43	-0.40	-0.40	-0.35	0.05
GM – S, L av.	0.14	0.15	0.07	0.13	
**CO_2/N_2**					
Short	-1.64	-1.80	-1.92	-1.68	0.24
Long	-0.39	-0.48	-0.42	-0.48	0.06
S, L av.	-1.02	-1.14	-1.17	-1.08	0.09
GM	-0.83	-1.06	-0.89	-0.99	0.10
AM	-1.33	-1.61	-1.47	-1.58	0.11
GM – S, L av.	0.19	0.07	0.28	0.09	
**CO_2/CH_4**					
Short	-1.14	-1.45	-1.48	-1.07	0.41
Long	-0.11	-0.28	-0.09	-0.22	0.13
S, L av.	-0.62	-0.87	-0.78	-0.65	0.14
GM	-1.02	-1.39	-1.14	-1.24	0.10
GM/2	-0.51	-0.69	-0.57	-0.62	0.05
AM	-1.40	-1.75	-1.57	-1.68	0.11
GM – S, L av.	-0.40	-0.52	-0.36	-0.60	
GM/2 – S, L av.	0.11	0.17	0.21	0.02	

[a] Hybrid *MP2:DFT+D + ΔCCSD(T)* calculations

In this study, we use the isolated pair lateral interaction energies which is consistent with the idea of GCMC simulations on a lattice of sites and with the mean field approximation. Let us assume that three calculations for the periodic systems are performed with all sites occupied with A-type molecules, with B-type molecules, or alternating with A and B type molecules yielding the energies *E*(A-A), *E*(B-B) and *E*(A-B). A two-body expansion yields

$$E(\text{A-A}) = 2E(\text{S-A})/\text{A-A} + E(\text{A-A})/\text{A-A} \tag{S19}$$

$$E(\text{A-B}) = E(\text{S-A})/\text{A-B} + E(\text{S-B})/\text{A-B} + E(\text{A-B})/\text{A-B} \tag{S20}$$

Where, e.g., *E*(S-A)/A-A is the two-body contribution for the binding of A onto the surface site S to the energy *E*(A-A), and *E*(A-B)/A-B is the energy contribution of the A···B pair interaction to *E*(A-B).

The arithmetic mean of the total energies *E*(A-A)/A-A and *E*(B-B)/B-B is

$$\begin{aligned}&\frac{1}{2}\left[E(\text{A-A}) + E(\text{B-B})\right] = \\ &\frac{1}{2}\left[E(\text{A-A})/\text{A-A} + E(\text{B-B})/\text{B-B}\right] + E(\text{S-A})/\text{A-A} + E(\text{S-B})/\text{B-B}\end{aligned} \tag{S21}$$

and the mixing energy becomes

$$\Delta E_{\text{mix}} = E(\text{A-B})/\text{A-B} - \frac{1}{2}\left[E(\text{A-A})/\text{A-A} + E(\text{B-B})/\text{B-B}\right] + \Delta \tag{S22}$$

$$\Delta = E(\text{S-A})/\text{A-B} - E(\text{S-A})/\text{A-A} + E(\text{S-B})/\text{A-B} - E(\text{S-B})/\text{B-B} \tag{S23}$$

There is an extra term Δ originating from the change of the molecule-surface interactions in the A···A (or B···B) and A···B systems.

Anisotropy factor. Table S4 shows the anisotropy factors calculated according to Eq. (29) in the main text

$$f_{\mathrm{AB}}(\mathrm{comput}) = \frac{\left|E_{\mathrm{AB}}^{\mathrm{av}}(\mathrm{comput})\right|}{\sqrt{\left|E_{\mathrm{AA}}^{\mathrm{av}}(\mathrm{comput})\right| \cdot \left|E_{\mathrm{BB}}^{\mathrm{av}}(\mathrm{comput})\right|}} \tag{S24}$$

for isolated interaction pairs with two different "comput." methods, CCSD(T)/CBS and PBE+D2/QZVP, and the resulting mixed pair interaction terms $-f_{\mathrm{AB}}L_{\mathrm{AB}}^{\mathrm{GM}}RT$ and mixing energies $-RT \cdot \Delta L_{\mathrm{mix}}^{\mathrm{fGM}}$.

Table S4. Anisotropy factors calculated with computed average lateral interaction energies. Comparison of anisotropy factor corrected and GM approximated mixed pair lateral interaction energy parameter $(-RT \cdot L_{\mathrm{AB}}^{\mathrm{GM}})$ and ab initio average lateral interaction energy ($E_{\mathrm{AB}}^{\mathrm{av}}$(ab-initio)). Energies are in kJ/mol while anisotropy factors have no unit.

A/B	CO_2/N_2	CO_2/N_2	CO_2/CH_4	CO_2/CH_4
f_{AB}	1.09 [a]	1.08 [b]	0.52 [a]	0.63 [b]
$-RT \cdot \Delta L_{\mathrm{mix}}^{\mathrm{GM}}$ [c]	0.58	0.58	0.47	0.47
$-f_{\mathrm{AB}}L_{\mathrm{AB}}^{\mathrm{GM}}RT$ [c]	-1.01	-1.00	-0.58	-0.70
$-RT \cdot \Delta L_{\mathrm{mix}}^{\mathrm{fGM}}$ [c]	0.50	0.51	1.00	0.88
$\Delta E_{\mathrm{mix}}(\mathrm{comput})$	0.50 [a]	0.47 [b]	1.04 [a]	0.88 [b]
$E_{\mathrm{AB}}^{\mathrm{av}}$(ab initio) [a]	-1.08	-1.08	-0.65	-0.65

[a] CCSD(T) calculations for isolated pairs. [b] PBE+D2/def2-QZVP calculations for isolated pairs. [c] Calculated from parameters for each gas obtained from linear MF fitting of pure gas isotherms.

S5. Ideal Adsorption Solution Theory (IAST)

Ideal adsorption solution theory (IAST)[12, 13] is the most widely used model for prediction of co-adsorption from pure gas adsorption isotherms. It assumes that the adsorbed phase behaves like an ideal solution of the adsorbed components. Consequently, the equilibrium between the adsorbed phase and gas phase can be described analogously to Raoult's law, according to which the partial vapor pressure of a gas component c, P_c, in an ideal liquid mixture can be calculated from the composition of the liquid mixture, x_c,

$$P_c = x_c P_c{}^{\circ} = y_c P \tag{S25}$$

where the vapor pressure of the pure liquid is $P_c{}^{\circ}$. The partial pressure can also be expressed as a product of gas phase composition (y_c) and total pressure (P).

The spreading pressure of a gas component c can be calculated by the following equation,

$$\pi_c(P_c{}^{\circ}) = \frac{RT}{A} \int_0^{P_c{}^{\circ}} \theta_c^{\text{pure}}(P)\, d\ln P \tag{S26}$$

where A is the area of the surface. π_c and $\theta_c^{\text{pure}}(P)$ are the spreading pressure and the pure gas adsorbed amount of component c, respectively. The equilibrium between the adsorbed components is attained when all the pure components have the same spreading pressure that is equal to the spreading pressure of the adsorbed mixture (π) itself. Considering a binary mixture (c = 1 or 2) this can be written as,

$$\pi = \pi_1\left(P_1{}^{\circ}\right) = \pi\left(P_2{}^{\circ}\right) \tag{S27}$$

The equilibrium pressures of the individual components, $P_c{}^{\circ}$, which are unknown, can be determined from the known composition of the gas (y_c) and the total pressure (P) by applying Eq. (S25).

$$P_c{}^{\circ} = \frac{y_c P}{x_c} \tag{S28}$$

To simplify the notation, let x and y represent the adsorbed and gas phase mole fraction of the first component (c = 1), respectively. Substituting the expressions of π and P° from Eqs. (S27) and (S28), respectively, in Eq.(S26), we obtain,

$$\int_0^{\frac{yP}{x}} \theta_1^{\mathrm{pure}}(P)\, d\ln P = \int_0^{\frac{(1-y)P}{(1-x)}} \theta_2^{\mathrm{pure}}(P)\, d\ln P \tag{S29}$$

For a binary mixture, the IAST problem reduces to finding a solution for x in the above equation. The integrations are performed on a set of data points representing the adsorbed amount θ_c^{pure} of the pure gas c at different pressures. Here, we adopted the most widely used approach where the isotherm data points of pure gas are fitted with an analytical model and then the integrals are evaluated analytically.[14] Since integration cannot be performed on a self-consistent equation, Eq. (S8), we used the non-linear mean-field isotherm equation,[1] Eq. (19) and (20), for fitting our single component data points obtained from the *ab initio* lattice GCMC simulations.

After obtaining the adsorbed phase composition the total adsorbed amount in the mixture, θ, is calculated according to the following equation,

$$\frac{1}{\theta} = \frac{x_1}{\theta_1^\circ} + \frac{x_2}{\theta_2^\circ} \tag{S30}$$

where θ_c° is the amount adsorbed in the pure gas isotherm at the same spreading pressure and temperature as that of the adsorbed mixture. The amount adsorbed for each component in the mixture is,

$$\theta_c = x_c\theta \tag{S31}$$

S6. Expansion of MF equilibrium constant for pure gas in continued fraction representation

Nonlinear mean-field (MF)

$$\theta_{\mathrm{A}}^{\mathrm{pure}} = \frac{K_{\mathrm{A}}^{*} P \exp\left[L_{\mathrm{AA}}^{*} \dfrac{K_{\mathrm{A}}^{*} P}{1 + K_{\mathrm{A}}^{*} P} \right]}{1 + K_{\mathrm{A}}^{*} P \exp\left[L_{\mathrm{AA}}^{*} \dfrac{K_{\mathrm{A}}^{*} P}{1 + K_{\mathrm{A}}^{*} P} \right]} \tag{S32}$$

Nonlinear mean-field (MF) with continued fraction representation

$$\theta_{\mathrm{A}}^{\mathrm{pure}} = \frac{K_{\mathrm{A}}^{*} P \exp\left[L_{\mathrm{AA}}^{*} \dfrac{K_{\mathrm{A}}^{*} P \exp\left(L_{\mathrm{AA}}^{*} K_{\mathrm{A}}^{*} P \big/ 1 + K_{\mathrm{A}}^{*} P \right)}{1 + K_{\mathrm{A}}^{*} P \exp\left(L_{\mathrm{AA}}^{*} K_{\mathrm{A}}^{*} P \big/ 1 + K_{\mathrm{A}}^{*} P \right)} \right]}{1 + K_{\mathrm{A}}^{*} P \exp\left[L_{\mathrm{AA}}^{*} \dfrac{K_{\mathrm{A}}^{*} P \exp\left(L_{\mathrm{AA}}^{*} K_{\mathrm{A}}^{*} P \big/ 1 + K_{\mathrm{A}}^{*} P \right)}{1 + K_{\mathrm{A}}^{*} P \exp\left(L_{\mathrm{AA}}^{*} K_{\mathrm{A}}^{*} P \big/ 1 + K_{\mathrm{A}}^{*} P \right)} \right]} \tag{S33}$$

S7. References

1. K. Sillar, A. Kundu and J. Sauer, *J. Phys. Chem. C*, 2017, **121**, 12789-12799.

2. R. H. Fowler and E. A. Guggenheim, *Statistical Thermodynamics: A Version of Statistical Mechanics for Students of Physics and Chemistry*, Cambridge University Press, London, 1956.

3. O. Redlich and J. N. S. Kwong, *Chem. Rev.*, 1949, **44**, 233-244.

4. A. Kundu, K. Sillar and J. Sauer, *J. Phys. Chem. Lett.*, 2017, **8**, 2713-2718.

5. A. R. Leach, *Molecular Modelling: Principles and applications*, Addison Wesley Longman Limited, England, 1996.

6. G. H. Hudson and J. C. McCoubrey, *Trans. Faraday Soc.*, 1960, **56**, 761-766.

7. *CRC Handbook of Chemistry and Physics*, CRC Press1993-94.

8. P. Atkins and J. D. Paula, *Physical Chemistry*, Oxford University Press2006.

9. S. G. Lias, in *NIST Chemistry WebBook, NIST Standard Reference Database Number 69*, eds. P. J. Linstrom and W. G. Mallard, National Institute of Standards and Technology, Gaithersburg MD2015.

10. K. Sillar and J. Sauer, *J. Am. Chem. Soc.*, 2012, **134**, 18354-18365.

11. A. Kundu, G. Piccini, K. Sillar and J. Sauer, *J. Am. Chem. Soc.*, 2016, **138**, 14047-14056.

12. A. L. Myers and J. M. Prausnitz, *AIChE J.*, 1965, **11**, 121-127.

13. K. S. Walton and D. S. Sholl, *AIChE J.*, 2015, **61**, 2757-2762.

14. C. M. Simon, B. Smit and M. Haranczyk, *Comput. Phys. Commun.*, 2016, **200**, 364-380.
